# Structural basis of cotranslational protein N-terminal acetylation by NatB in human cells

**DOI:** 10.1038/s41467-026-75207-1

**Published:** 2026-07-11

**Authors:** Natalia Silva Alves, Pawel Knejski, Alain Scaiola, Marc Leibundgut, Martin Gamerdinger, Nenad Ban, Elke Deuerling

**Affiliations:** 1 Department of Biology, Molecular Microbiology, University of Konstanz, Konstanz, Germany; 2 Department of Biology, Institute of Molecular Biology and Biophysics, ETH Zurich, Zurich, Switzerland

**Keywords:** X-ray crystallography, Protein folding, Enzyme mechanisms, Acetylation, Acetyltransferases

## Abstract

Cotranslational N-terminal acetylation is a widespread modification that shapes protein stability, localization, and function in eukaryotic cells. The essential human NatB complex (NAA25-NAA20) acetylates the initiator methionine of a substantial fraction of the proteome, yet how NatB engages translating ribosomes has remained unclear. Here we define the cotranslational mechanism underlying NatB function. NatB is recruited by the nascent polypeptide-associated complex (NAC) through a high-affinity interaction between the NACα UBA domain and the auxiliary subunit NAA25, while both NatB subunits form additional contacts with the ribosomal surface near the tunnel exit. Together, these interactions position the NatB active site directly adjacent to the emerging nascent chain, enabling efficient modification of newly synthesized proteins. Structural comparisons reveal a conserved ribosome-binding architecture shared with other N-acetyltransferases, including NatA/E and NatD, implying mutually exclusive ribosome occupancy. Together with prior work, these findings establish NAC as a central organizer of cotranslational N-terminal processing.

## Abstract (web summary)

N-terminal acetylation is one of the most common protein modifications. Here, the authors show how NAC recruits the essential enzyme NatB to translating ribosomes, revealing a general mechanism for cotranslational protein modification.

## Introduction

Protein synthesis in eukaryotes is tightly coupled to cotranslational modification of the nascent N terminus. Approximately 80–90% of cytonuclear proteins undergo irreversible cotranslational N-terminal processing by a set of specialized ribosome-associated enzymes. These include methionine aminopeptidases (MetAPs), which remove the initiator methionine, as well as N-acetyltransferases (NATs) and N-myristoyltransferases (NMTs), which attach an N-terminal (Nt) acetyl and myristoyl moiety, respectively, to the exposed α-amino group. Roughly 40% of the processed proteins undergo a two-step pathway—methionine excision followed by acetylation or myristoylation—whereas the remainder are directly acetylated on the initiator methionine. These irreversible modifications profoundly influence protein stability, folding, interactions, and membrane association, and their disruption is linked to cancer and neurodevelopmental diseases^1–12^.

Cotranslational N-terminal modification must occur within a narrow temporal window, immediately after the nascent chain emerges from the ribosomal tunnel and before folding begins^3,13,14^. How multiple cotranslational enzymes can act efficiently without interference at the ribosomal tunnel exit has long remained unclear. Recent work has shown that two-step N-terminal processing pathways are coordinated by the nascent polypeptide-associated complex (NAC), a conserved heterodimer (NACα and NACβ) that occupies the ribosomal exit site of virtually all translating ribosomes^15–19^. Through flexible C-terminal extensions, NAC recruits MetAP1 together with downstream NATs (NatA/E, NatD) or NMTs (NMT1, NMT2) to translating ribosomes, thereby organizing sequential modification of nascent N termini^19–27^.

In parallel, a large fraction of nascent proteins must undergo direct acetylation of the initiator methionine^5^. Most of these substrates are modified by NatB, a heterodimer composed of the auxiliary subunit NAA25 and the catalytic subunit NAA20, which targets proteins with acidic or polar residues at position two following the initiator methionine (D, E, N, or Q)^28–30^. Substrate recognition is primarily dictated by the first two residues of the nascent chain. The initiator methionine is accommodated within a hydrophobic pocket of NAA20, while the second residue engages in specific hydrogen-bonding interactions that define substrate specificity^28^. NatB is essential for viability in metazoans^31–33^ and acetylates approximately 20% of the human proteome, including key cytoskeletal and regulatory proteins, such as actin, tropomyosin, CDK2, and α-synuclein^30,34–36^. Pathogenic variants in NAA20 have been identified in individuals with developmental delay, intellectual disability, and microcephaly^37^.

Despite its importance, how human NatB engages translating ribosomes and how its activity is coordinated with NAC-dependent two-step modification pathways remains unknown. In this study, we define the cotranslational mechanism of human NatB, elucidate how it is recruited to ribosomes, and uncover how its action is integrated with NAC and other N-acetyltransferases at the ribosomal tunnel exit.

## Results

### NAC promotes cotranslational Nt-acetylation of initiator methionines by NatB

Recent work has established NAC as an organizer of ribosome-associated multi-enzyme assemblies that ensure the correct modification of newly exposed N termini following initiator methionine excision by MetAP1^19^. In addition to these substrates, a substantial fraction of nascent proteins (~20%) undergo direct Nt-acetylation of the initiator methionine by NatB^30^. Yet, how human NatB interacts with translating ribosomes to execute this cotranslational reaction has remained unclear.

**Fig. 1:**
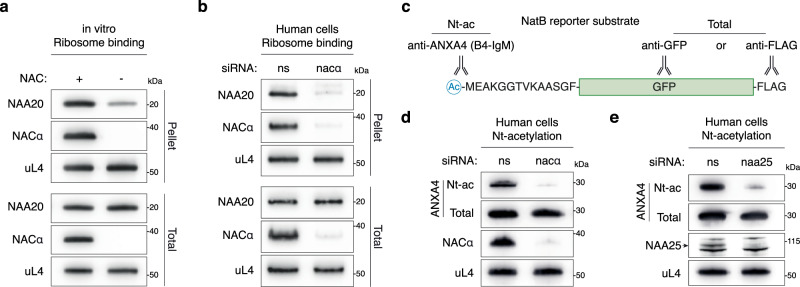
NAC facilitates cotranslational protein Nt-acetylation by NatB. **a** In vitro sucrose cushion ultracentrifugation of purified ribosomes and NatB in the presence and absence of purified NAC. Ribosomal pellet and total fractions were analyzed by immunoblotting. NatB was detected using antibodies against NAA20. One representative experiment of three independent biological replicates is shown. **b** Ribosome association of NatB after siRNA-mediated knockdown of NACα in human HEK293T cells. Total cellular ribosomes were pelleted from cytosolic fraction by sucrose cushion ultracentrifugation. Proteins in total and ribosomal pellet fractions were then detected by immunoblotting. One representative experiment of three independent biological replicates is shown. **c** Schematic of NatB reporter substrate comprising the first 14 amino acids of mouse ANXA4 fused to GFP-FLAG. Nt-acetyl-specific ANXA4 B4-IgM^38^ and GFP or FLAG antibodies are used to detect Nt-acylated (Nt-ac) and total reporter levels, respectively. **d**, **e** Nt-acetylation of NatB reporter substrate ANXA4 indicated in panel c after siRNA-mediated knockdown of NACα (**d**) and NAA25 (**e**) in human HEK293T cells. Protein levels were detected by immunoblotting. One representative experiment of three independent biological replicates is shown. Source data are provided as a Source Data file.

Given that NAC facilitates ribosome binding of other NATs, including NatA/E and NatD^22,24^, we asked whether NatB might similarly depend on NAC for productive ribosome engagement. In vitro ribosome-binding assays using purified components revealed that NatB associates efficiently with ribosomes in the presence of NAC, whereas only weak binding was observed in its absence (Fig. 1a). These data indicate that NAC facilitates the recruitment or stabilization of NatB on translating ribosomes.

Consistent with this, knockdown of NAC by RNA interference (RNAi) markedly reduced the ribosome association of NatB in human cells (Fig. 1b). Moreover, in agreement with impaired ribosomal recruitment, the Nt-acetylation of a NatB reporter substrate—comprising the first 14 amino acids of ANXA4 fused to GFP-FLAG (Fig. 1c)^38^—was strongly diminished upon NAC depletion (Fig. 1d), similar to knockdown of the NatB subunit NAA25 (Fig. 1e). Thus, beyond its established role in coordinating MetAP1-NatA/E- and MetAP1-NatD-dependent pathways, NAC also promotes efficient cotranslational Nt-acetylation of initiator methionines by NatB.

### Structure of the human NAC-NatB complex on translating ribosomes

**Fig. 2:**
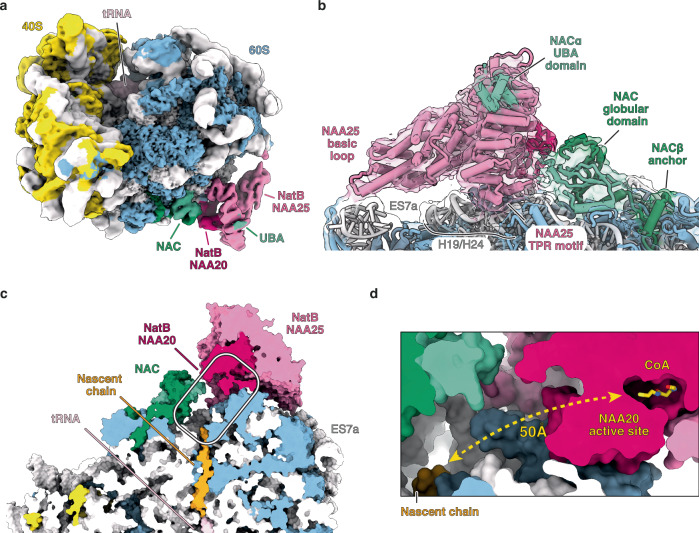
Cryo-EM map of RNC^ANXA4^ in complex with NAC and NatB. **a** Overview of the cryo-EM map of the RNC^ANXA4^ **·** NAC **·** NatB complex locally filtered according to resolution, with key domains indicated in colors. Ribosomal proteins of the small and large subunits are shown in yellow and light blue, respectively, rRNA in white, NACα in light green, NACβ in green, NAA25 in pink, NAA20 in deep magenta, and tRNA in light pink. **b** Association of NatB with the ribosomal surface in proximity to the peptidyl tunnel exit, highlighting its recruitment via the NAC UBA domain. The RNC^ANXA4^ **·** NAC **·** NatB model is fitted into a cryo-EM map filtered to 5 Å. α-helices are depicted as cylinders, β-sheets as arrows, and bases as stubs. **c** Cross section of the RNC^ANXA4^ **·** NAC **·** NatB complex highlighting the nascent chain (orange) within the ribosomal peptidyl tunnel. The rectangle indicates the region surrounding the tunnel exit. **d** Close up of peptidyl tunnel exit showing the distance the nascent chain must span to reach the active site of NAA20 along a straight trajectory, consistent with other early-acting cotranslational enzymes. Coenzyme A in the active site (yellow) is shown in atom-and-bond representation.

To define how NatB is positioned on the ribosome and gains access to nascent substrates, we determined the structure of NatB bound to a stalled ribosome-nascent chain complex (RNC) translating the NatB substrate ANXA4 (RNC^ANXA4^), together with NAC, using single-particle cryo-electron microscopy (cryo-EM). The complex was reconstituted using a stalled human RNC carrying the N terminus of mouse ANXA4 followed by a modified Xbp1u arrest peptide^39^, yielding a 60-residue nascent chain. Purified RNCs were incubated with recombinant human NAC and NatB in the presence of Coenzyme A (CoA) instead of acetyl-CoA to allow substrate engagement while preventing catalysis. The reconstruction reached an overall resolution of 3.19 Å, with local resolutions of 4–10 Å for NAC and NatB, allowing reliable docking of known structures (Fig. 2, Supplementary Fig. 1 and 2).

The cryo-EM map revealed that NAC and NatB simultaneously bind to the tunnel exit region of the translating ribosome (Fig. 2a). The high-affinity ribosomal anchor of the NACβ N terminus engaged the ribosome at the same site on eL19 and eL22 as observed in previous structures^40^, while the NAC globular domain was docked adjacent to the tunnel exit, similar to its configuration in complexes with MetAP1, NatA/E, or NatD (Fig. 2b)^21,22,24^.

NatB is positioned immediately next to the NAC globular domain, with the catalytic center of its NAA20 subunit oriented toward the tunnel exit where the nascent chain emerges (Fig. 2c). Density corresponding to the nascent peptide within the substrate-binding pocket was not resolved, likely reflecting conformational flexibility. The active site is localized approximately 50 Å from the tunnel exit (Fig. 2d), consistent with cotranslational processing of nascent chains as soon as they reach ~50 amino acids in length. This geometry parallels that of other early-acting cotranslational enzymes, including MetAP1, NatD, and NMTs^20,21,24^, and contrasts with NatA/E, which engage substantially longer nascent chains^22^.

### Multivalent ribosome docking of NatB via NAA25 and NAA20

**Fig. 3:**
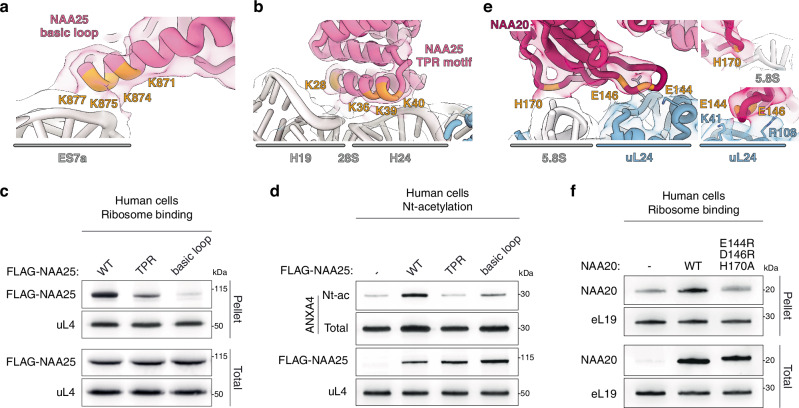
Ribosome interaction mechanism of NatB. **a** Close-up of the binding interface between the NAA25 basic loop and ES7a. The conserved poly-lysine stretch is highlighted in orange; NAA25 is shown in pink, ribosomal proteins in blue, and rRNA in white, with bases depicted as stubs. **b** Close-up of the binding interface between NAA25 TPR motif residues and helices H19 and H24 of 28S rRNA. Mutated lysine residues interacting with rRNA are highlighted in orange; NAA25 is shown in pink, ribosomal proteins in blue, and rRNA in white, with bases depicted as stubs. **c** Ribosome association of indicated FLAG-tagged NAA25 variants in human HEK293T cells in the endogenous NAA25 siRNA knockdown background. Cytosolic fraction of cells was layered onto a sucrose cushion and ribosomes pelleted by ultracentrifugation. Proteins in total and ribosomal pellet fractions were then detected by immunoblotting. Ectopically expressed NAA25 variants were detected using anti-FLAG antibodies. One representative experiment of three independent biological replicates is shown. **d** Nt-acetylation (Nt-ac) of NatB reporter substrate ANXA4 (Fig. 1c) in HEK293T cells expressing the indicated FLAG-tagged NAA25 variants in the endogenous NAA25 siRNA knockdown background. Protein levels were detected by immunoblotting. Ectopically expressed NAA25 variants were detected using anti-FLAG antibodies. One representative experiment of three independent biological replicates is shown. **e** Close-up of the binding interface between the NAA20 C-terminal loops and 5.8S rRNA. Mutated residues involved in interactions with rRNA and uL24 are highlighted in orange; NAA20 is shown in deep magenta, ribosomal proteins in blue, and rRNA in white. **f** Ribosome association of indicated NAA20 variants in human HEK293T cells in the endogenous NAA20 siRNA knockdown background. Cytosolic fraction of cells was layered onto a sucrose cushion and ribosomes pelleted by ultracentrifugation. Proteins in total and ribosomal pellet fractions were then detected by immunoblotting. One representative experiment of three independent biological replicates is shown. Source data are provided as a Source Data file.

The cryo-EM map showed that ribosome contacts by NatB are mediated primarily by its auxiliary subunit, NAA25, through electrostatic interactions with ribosomal RNA (rRNA) segments. On one hand, a conserved basic loop in the C-terminal region of NAA25 (K871-K877) contacts rRNA expansion segment ES7a, a flexible element located distal to the tunnel exit (Figs. 2c, 3a, and below). A second contact, positioned closer to the tunnel exit, is formed by an N-terminal tetratricopeptide repeat (TPR) motif of NAA25, where several basic residues (K28, K36, K39, and K40) interact with 28S rRNA helices H19 and H24 adjacent to ribosomal protein uL24 (Figs. 2c, 3b, and below).

The identified positively charged ribosome-interacting residues are conserved across metazoans (Supplementary Fig. 3), suggesting functional relevance. To test this, we introduced charge-reversal mutations disrupting either the TPR interface (NAA25 K28E/K36E/K39E/K40E) or the basic loop (NAA25 K871E/K874E/K875E/K877E). Both mutations substantially reduced NatB ribosome association in human cells despite normal NAC expression (Fig. 3c). Correspondingly, Nt-acetylation of the ANXA4 reporter was also strongly impaired in cells expressing the NAA25 mutants (Fig. 3d). Notably, the TPR mutant produced the most severe Nt-acetylation defect, highlighting its importance of positioning NatB proximal to the tunnel exit (at rRNA helices H19 and H24, Fig. 2b, c) for efficient capture of nascent N termini.

Unexpectedly, the structure further revealed direct ribosome contacts made by the catalytic subunit NAA20. Conserved residues in the C-terminal region of NAA20 (Supplementary Fig. 3) contacted 5.8S rRNA and uL24, adjacent to the NAA25 TPR interface (Fig. 3e). Disruption of this NAA20 contact by structure-guided mutations (NAA20 E144R/D146R/H170A) diminished ribosome binding of NatB in cells (Fig. 3f).

Together, these findings show that human NatB engages translating ribosomes through three independent interfaces involving both the auxiliary and catalytic subunits. This multivalent docking mechanism distinguishes NatB from other multi-subunit NATs, which bind exclusively via the auxiliary subunit^22^, and underscores how precise spatial organization at the tunnel exit enables efficient cotranslational Nt-acetylation of initiator methionines.

### The NACα UBA domain recruits NatB to translating ribosomes

Although NatB and the NAC globular domain bind at adjacent sites at the ribosomal tunnel exit, they do not directly contact each other, suggesting that NAC stabilizes NatB on ribosomes through a distinct interface. Consistent with this idea, an additional globular density was observed on the surface of the NatB auxiliary subunit NAA25 (Fig. 2a). This density closely resembles the three-helix bundle characteristic of the UBA domain located at the tip of the NACα C-terminal arm^19^.

**Fig. 4:**
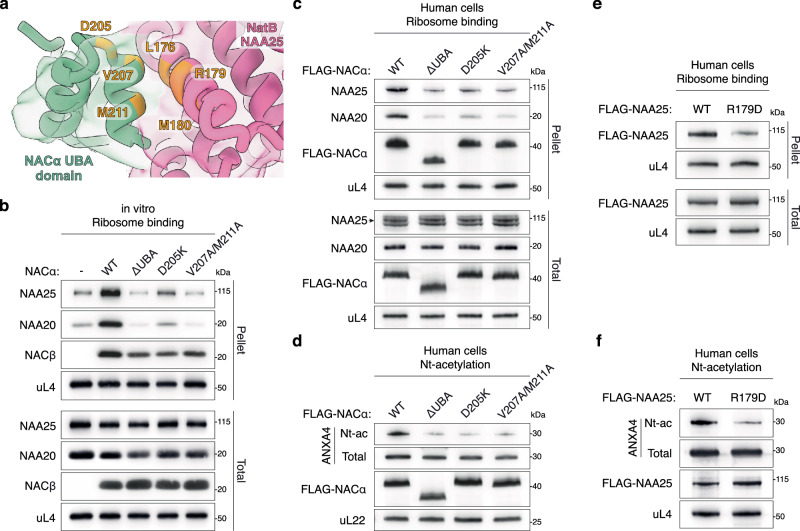
NACα UBA domain recruits NatB to translating ribosomes. **a** Close-up view of the binding interface between the NACα UBA domain (green) and the NAA25 auxiliary subunit TPR motif (pink) with interacting residues highlighted in orange. The model is shown as cartoon and fitted into the cryo-EM map filtered to 5 Å resolution. **b** In vitro sucrose cushion ultracentrifugation of purified ribosomes and NatB in the presence and absence of indicated NAC variants. Ribosomal pellet and total fractions were analyzed by immunoblotting. One representative experiment of three independent biological replicates is shown. **c** Ribosome association of NatB in human HEK293T cells expressing indicated FLAG-tagged NACα variants in the endogenous NACα siRNA knockdown background. Cytosolic fraction of cells was layered onto a sucrose cushion and ribosomes pelleted by ultracentrifugation. Proteins in total and ribosomal pellet fractions were then detected by immunoblotting. Ectopically expressed NACα variants were detected using anti-FLAG antibodies. One representative experiment of three independent biological replicates is shown. **d** Nt-acetylation (Nt-ac) of NatB reporter substrate ANXA4 (Fig. 1c) in cells as described in (**c**). Ectopically expressed NACα variants were detected using anti-FLAG antibodies. One representative experiment of three independent biological replicates is shown. **e** Ribosome association of indicated FLAG-tagged NAA25 variants in human HEK293T cells in the endogenous NAA25 siRNA knockdown background. Cytosolic ribosomes were pelleted by sucrose cushion ultracentrifugation. Proteins in total and ribosomal pellet fractions were then detected by immunoblotting. One representative experiment of three independent biological replicates is shown. **f** Nt-acetylation (Nt-ac) of NatB reporter substrate ANXA4 (Fig. 1c) in cells as described in (**e**). Protein levels were detected by immunoblotting. One representative experiment of three independent biological replicates is shown. Source data are provided as a Source Data file.

To define this interaction, we used AlphaFold^41^ to model the NatB-NAC complex. Strikingly, the predicted position and conformation of the NACα UBA domain matched the extra density observed on NAA25 in the cryo-EM map (Fig. 4a), suggesting that NatB is recruited by NAC via the same UBA domain previously shown to engage NatA/E and NatD^22,24^. Thus, NAC appears to use a common recruitment module to coordinate multiple NATs at the ribosome.

Guided by the AlphaFold model, we generated mutants designed to selectively disrupt the predicted NACα UBA-NAA25 binding interface. This interface involved both electrostatic and hydrophobic interactions (Fig. 4a). Charge-reversal mutations were introduced in either NACα (D205K) or NAA25 (R179D) to disrupt a predicted salt bridge between NAA25 R179 and NACα D205, while hydrophobic contacts were perturbed by alanine substitutions of conserved residues V207 and M211 of NACα (V207A/M211A), predicted to engage NAA25 L176 and M180. NAA25 variants harboring mutations at these hydrophobic residues were unstable and could therefore not be investigated. In addition, we generated a deletion mutant lacking the entire UBA domain of NACα (Δ176-215, referred to as ΔUBA).

In vitro ribosome-binding assays using purified components showed that all three NACα mutations (D205K, V207A/M211A, and ΔUBA) almost completely abolished the ability of NAC to stabilize NatB on ribosomes (Fig. 4b). Consistent with the in vitro data, NatB ribosome association was also strongly reduced in cells expressing these NACα mutants (Fig. 4c). Moreover, functionally, all of the mutants were defective in promoting the Nt-acetylation of the ANXA4 reporter in NACα knockdown cells (Fig. 4d). Impairment in ribosome association and Nt-acetylation was also observed in cells expressing NatB carrying the charge-reversal mutation at the NACα UBA-binding interface (NAA25 R179D) (Fig. 4e, f).

Together, these data establish the NACα UBA domain as the key recruitment module for NatB on translating ribosomes. Thus, the UBA domain of NACα coordinates the cotranslational activities of at least four distinct N-acetyltransferases—the dual composite enzyme NatA/E, NatB, and NatD—ensuring efficient N-terminal acetylation across nearly the entire spectrum of cotranslational NAT substrates^22,24^.

### Comparison of ribosome interaction mechanisms of NatA/E, NatB, and NatD

**Fig. 5:**
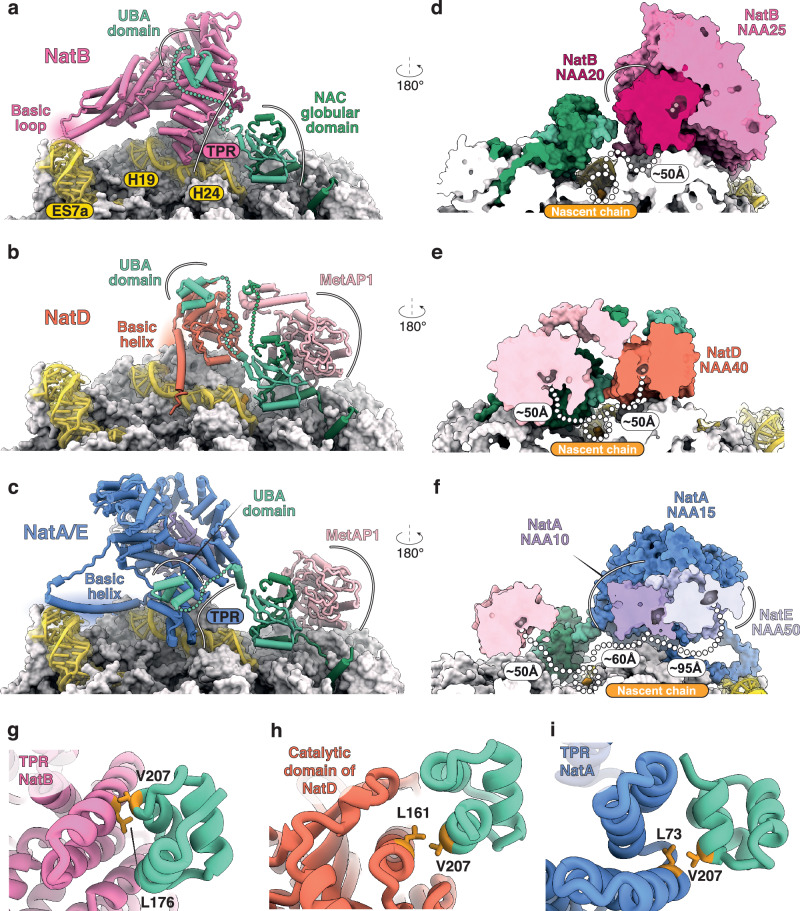
NatB, NatA/E, and NatD compete for a common ribosomal docking site. **a–c** Structural comparison of ribosome-bound NatB (**a**; this study), NatD (**b**; PDB: 9SYR^24^), and NatA/E (**c**; PDB: 9F1C^22^) in complex with NAC. All NATs bind an overlapping platform on 28S rRNA helices H19 and H24 near the tunnel exit. NatB and NatA/E engage the ribosome via TPR motifs, whereas NatD uses a basic helix; NatB and NatA/E additionally contact ES7a. Ribosomal contact sites are highlighted (yellow). The ribosome is shown as a white surface; NACα and NACβ in shades of green, NatB in pink, NatD in red, NatA/E in blue, and MetAP1 in pale pink. Unresolved NAC regions are indicated by dotted lines. **d–f** Backside views of the complexes shown as surface representation. The cut sections reveal the active sites of NatB (**d**), NatD and MetAP1 (**e**), and NatA/E and MetAP1 (**f**). NAA20 is shown in deep magenta, the nascent chain in orange, NAA10 and NAA50 in shades of purple. Dotted lines indicate the trajectory required for the nascent chain to reach each active site; distances were measured from the last visible residue to catalytic sites of enzymes. **g–i** Close-up views show the NACα UBA domain contacting NatB (**g**), NatD (**h**), and NatA/E (**i**). NACα residue V207 participates in all interfaces and aligns with a conserved leucine in each enzyme.

The ribosome interaction mode of human NatB revealed here closely parallels that of NatA/E and NatD (which bind together with MetAP1^22,24^), while incorporating distinct, enzyme-specific features. All three NATs bind to a common site adjacent to the NAC globular domain at the tunnel exit, positioning their catalytic centers to engage nascent N termini cotranslationally (Fig. 5d–f). In both multi-subunit NATs, NatA/E and NatB, ribosome association is mediated primarily by their auxiliary subunits, which contact the distal rRNA expansion segment ES7a. In NatA/E, this interaction is formed by a long basic helix, whereas in NatB, it is mediated by a flexible basic loop (Fig. 5a, c). In addition, both complexes share a second contact near the tunnel exit through basic residues in N-terminal TPR elements that engage 28S rRNA helices H19 and H24 (Fig. 5a, c).

A key distinction of NatB is the additional ribosome contact contributed by its catalytic subunit NAA20 near the tunnel exit (Figs. 2c, 3e, and 5d). By contrast, the catalytic subunits of NatA/E (NAA10 and NAA50) are positioned further from the exit, consistent with the requirement for MetAP1 and NMTs to access the nascent N terminus before NatA/E^20^. NatD, a single-subunit enzyme, does not engage ES7a but also binds H19 and H24, using a short N-terminal basic helix rather than a TPR motif (Fig. 5b). Thus, rRNA helices H19 and H24 emerge as a shared docking platform for all three NATs, underscoring their central importance for cotranslational Nt-acetylation. This conclusion is supported by the particularly severe acetylation defects observed upon disruption of this interface in NatB (Fig. 3d).

The broadly similar ribosome binding modes of NatA/E, NatB, and NatD (with an estimated cellular concentration of ~0.65 µM, ~0.14 µM, and ~0.12 µM, respectively^42^) suggest that they compete for access to translating ribosomes. Consistent with this idea, in vitro binding experiments show that NatB can be displaced by the other two NATs from ribosomes, indicating that only one NAT occupies a ribosome at a given time (Supplementary Fig. 4). These findings imply that the different NATs rapidly and repeatedly sample translating ribosomes to gain timely access to their specific substrates.

This dynamic scanning is likely facilitated by long-range tethering through the UBA domain at the C-terminus of NACα, a shared interaction observed for all three NATs. As shown here for NatB (Fig. 4) and previously for NatA/E and NatD^22,24^, deletion of the NACα UBA domain severely compromises cotranslational Nt-acetylation. Although UBA domains classically bind ubiquitin via a conserved hydrophobic surface^43^, the NACα UBA domain does not bind ubiquitin^44^ but instead uses an analogous hydrophobic patch to recruit multiple NATs. While the precise side-chain interactions differ among NatA/E, NatB, and NatD, cryo-EM and AlphaFold-guided analyses reveal a strongly overlapping binding interface, with residues, such as NACα V207 contributing to all three interactions (Fig. 5g–i). Together, these observations identify the NACα UBA domain as a versatile multi-adapter that coordinates competing N-acetyltransferases at the ribosome to ensure efficient cotranslational modification.

## Discussion

This study defines the molecular basis of cotranslational Nt-acetylation by human NatB and places this pathway within a broader organizational framework at the ribosomal exit site. The overall fold and domain arrangement of ribosome-bound NatB closely resembles that of previously determined ribosome-free structures of NatB^34,45^, with no major conformational differences. However, the ribosome-bound state resolves key regions—particularly those mediating ribosomal contacts—that were not well defined previously. We show that NatB is recruited to translating ribosomes by NAC, primarily through interactions between the UBA domain of NACα and the auxiliary NatB subunit NAA25. Together with direct electrostatic contacts of both NatB subunits with the ribosomal surface, this interaction positions NatB adjacent to the ribosomal tunnel, enabling efficient acetylation of initiator methionines as they emerge from the ribosome.

Comparison of the NatB recruitment mechanism with previously characterized architectures of NatA/E^22,25^ and NatD^24^ reveals an unexpected convergence. Despite acting on distinct substrates, all three NATs occupy overlapping ribosomal binding sites, engage the same rRNA helices near the tunnel exit (H19 and H24), and rely on a common hydrophobic surface within the NACα UBA domain for recruitment (Fig. 5). These similarities are striking given that NatB acetylates the initiator methionine directly, whereas NatA and NatD require prior methionine excision by MetAP1. Nonetheless, the shared binding modes imply that NAT engagement with the ribosome is mutually exclusive, raising the question of how accurate substrate selection is achieved in such a crowded environment.

Our findings support a model in which cotranslational enzymes rapidly and transiently sample nascent chains as they emerge from the tunnel. Specificity is not enforced at the level of ribosome binding, but rather by structural constraints within each enzyme’s active site. For NATs, the identity of the amino acids following the initiator methionine plays a decisive role in determining whether a nascent N terminus can productively engage the catalytic pocket. NatB, for example, acetylates the initiator methionine only when followed by acidic or polar residues, precluding modification of substrates destined for methionine excision^2,28^. Conversely, MetAP1 cannot cleave initiator methionines followed by bulky residues, preventing inappropriate processing of NatB substrates^46,47^. Notably, the MetAP1 docking site appears to remain accessible on ribosomes bound by NAC and NatB, suggesting that MetAP1 could co-occupy these complexes, as observed for NatA/E and NatD (Fig. 5), but would remain catalytically unproductive on NatB substrates. Thus, fidelity is ensured by substrate-encoded compatibility with enzyme active sites, allowing multiple enzymes to interrogate the same nascent chain without risk of misprocessing. A potential exception arises with NatA, which could in principle acetylate N-terminal glycines of NMT substrates after methionine excision by MetAP1^11,48^. However, the NatA active site lies further from the tunnel exit than those of other enzymes, including NMTs. This spatial arrangement allows NMTs to access nascent N termini first, thereby limiting premature NatA-mediated acetylation, as shown previously^19,20^.

Such rapid ribosome sampling requires that modifying enzymes be maintained at high local concentration near the ribosomal tunnel exit. NAC appears to fulfill this role by acting as a flexible scaffold. Selective ribosome profiling has shown that NAC is present on nearly all ribosomes at the moment the nascent N terminus emerges^16,17^. Through its extended C-terminal arms, NACα and NACβ recruit a broad repertoire of cotranslational enzymes, including MetAP1, NMT1, NMT2, NatA/E, NatB, and NatD^19–24^. In combination, these enzymes account for nearly all known N-terminal processing events in human cells^3^. The only enzyme whose ribosome-binding mechanism—and potential dependence on NAC—remains unclear is NatC, a trimeric complex (NAA35, NAA30, NAA38) that overlaps in substrate specificity with NatE^5,49,50^. In addition to these modifying enzymes, previous studies showed that NAC also recruits the cotranslational chaperone CHP1/PBDC1 and the ER-targeting factor SRP with its C-terminal tails^40,51,52^.

This organization suggests a shift from the traditional view of protein biogenesis factors engaging ribosomes through stochastic diffusion. Instead, NAC establishes a dynamic yet spatially constrained environment—a cloud of modifying enzymes, chaperones, and targeting factors poised at the tunnel exit—ensuring timely inspection and processing of nascent chains. This structured cytoplasmic microenvironment provides a unifying framework for understanding how cotranslational pathways achieve both efficiency and specificity at the earliest stages of protein biogenesis.

## Methods

### Protein purifications

For purification of human NatB (NAA25 + NAA20 complex), a URA3-selectable, galactose-inducible pYES2.1 bicistronic plasmid encoding human 6xHis-NAA25 and NAA20 was used to transform *Saccharomyces cerevisiae* strain BCY123. Individual transformants were screened for expression and stored as glycerol stocks at −80 °C.

For protein production, glycerol stocks were streaked onto uracil-dropout synthetic defined (SD-Ura) agar plates and incubated for 48 h at 30 °C. Five to ten colonies were inoculated into SD-Ura medium containing 2% glucose and grown overnight at 30 °C with shaking. The following day, an amount of overnight culture corresponding to an OD_600_ of 0.4 was transferred into SD-Ura medium supplemented with 2% raffinose and grown at 30 °C with shaking to an OD_600_ of ~1.6. Cultures were then diluted with SD-Ura medium containing 2% galactose to a final OD_600_ of 0.8 to induce protein expression. After overnight induction, cells were harvested and immediately frozen in liquid nitrogen.

Frozen cell pellets were converted into cell powder using a pre-cooled CryoMill (Retsch) at 30 Hz for 60 s. The resulting powder was thawed in an equal volume of lysis buffer (50 mM potassium phosphate pH 8.0, 500 mM NaCl, 10 mM β-mercaptoethanol, 10% glycerol) supplemented with protease inhibitors (8 µg/ml pepstatin, 10 µg/ml aprotinin, 5 µg/ml leupeptin, 2 mM AEBSF) and 5 mg DNase I. Lysates were cleared by centrifugation at 212,000 x g for 60 min at 4 °C in a Type 50.2 Ti fixed-angle rotor (Beckman Colter). His-tag affinity purification was performed using a gravity-flow Ni-IDA resin (Macherey-Nagel). After binding, the column was washed with lysis buffer containing 50 mM sodium phosphate (pH 8.0) and 2 mM β-mercaptoethanol, followed by a second wash with lysis buffer adjusted to 25 mM NaCl until the absorbance at 280 nm returned to baseline. Bound proteins were eluted with elution buffer (50 mM sodium phosphate pH 8.0, 120 mM NaCl, and 2 mM β-mercaptoethanol, 250 mM imidazole) and dialyzed overnight against dialysis buffer (20 mM sodium phosphate pH 7.4, 250 mM NaCl, 2 mM β-mercaptoethanol, 10% glycerol).

Further purification was achieved by ion-exchange chromatography. Dialyzed protein was first applied to a Mono Q 5/50 GL anion-exchange column (Cytiva), and the flow-through together with wash fractions was subsequently loaded onto a Capto HiRes S 10/100 cation-exchange column (Cytiva). Proteins were eluted using a 20-column-volume linear gradient from buffer A (20 mM sodium phosphate pH 7.4, 250 mM NaCl, 2 mM β-mercaptoethanol, 10% glycerol) to 90% buffer B (20 mM sodium phosphate pH 7.4, 1 M NaCl, 2 mM β-mercaptoethanol, 10% glycerol). Fractions containing NatB were pooled, dialyzed overnight into storage buffer (20 mM sodium phosphate pH 7.4, 25 mM NaCl, 2 mM β-mercaptoethanol, 5% glycerol, 6 mM MgCl_2_), flash-frozen in liquid nitrogen, and stored at −80 °C.

Human NAC (NACα/NACA + NACβ/BTF3b complex), NatD (NAA40), and NatA (NAA15 + NAA10 complex) were expressed as His-SUMO or His-TEV fusion proteins in *E. coli* BL21(DE3) and purified similarly as previously described^17,21^. In brief, cells were grown at 37 °C to OD_600_ ~ 1.5, shifted to 20 °C, and expression was induced with 1 mM IPTG for ~5 h. Cells were harvested and resuspended in lysis buffer (50 mM sodium phosphate pH 8.0, 300 mM NaCl, 6 mM MgCl_2_, 2 mM β-mercaptoethanol, 10% glycerol) supplemented with protease inhibitors and DNase I. Cells were lysed by high-pressure homogenization (EmulsiFlex C3), and lysates were clarified by centrifugation (2 × 20 min, 30,000 x g, 4 °C). His-tagged proteins were purified using Ni-IDA gravity columns (Macherey-Nagel), washed with high-salt buffer (lysis buffer containing 750 mM NaCl), and eluted with elution buffer (lysis buffer containing 250 mM imidazole). Eluted proteins were dialyzed (20 mM sodium phosphate pH 7.4, 25 mM NaCl, 6 mM MgCl_2_, 2 mM β-mercaptoethanol, 5% glycerol) overnight in the presence of SUMO protease (Ulp1, 8 µg/mg protein) to remove the fusion tag, followed by ion-exchange chromatography for further purification. Protein-containing fractions were pooled, dialyzed into storage buffer (20 mM sodium phosphate pH 7.4, 25 mM NaCl, 6 mM MgCl_2_, 2 mM β-mercaptoethanol, 10% glycerol), flash-frozen in liquid nitrogen, and stored at −80 °C.

Identity and purity of recombinant proteins generated in this study were tested by Coomassie staining (Supplementary Fig. 5) and immunoblot detection (Fig. 4b) using specific antibodies (see Antibodies section below).

Protein purification and biotinylation of anti-GFP nanobody were performed as previously described^24^. For purification of GADD34 Δ240, a vector containing cDNA encoding GADD34Δ240 protein^53^ was transformed into *E. coli* BL21(DE3) pLysS cells. Bacteria were grown overnight at 37 °C with shaking in LB medium supplemented with 50 µg/ml ampicillin. The culture was transferred to 6 L of TB medium supplemented with 50 µg/ml ampicillin and grown at 37 °C with shaking at 120 rpm. Protein expression was induced at OD_600_ of 0.95 by the addition of 1 mM IPTG, and cells were harvested after 2 h of expression by centrifugation.

Cell pellets were resuspended in resuspension buffer (500 mM KCl, 50 mM HEPES pH 7.5, 0.5 mM DTT, 40 mM imidazole) supplemented with protease inhibitors (10 µM bestatin, 1 µM pepstatin, 10 µM leupeptin, 100 µM PMSF, and 2 µM E-64). Cells were lysed by sonication using a Branson Sonifier (three cycles, 50% amplitude, 1 min total pulse time with 1 s pulses and 2 s intervals, with cooling between cycles). Insoluble material was removed by centrifugation at 30,000 x g for 45 min at 4 °C using an SS-34 fixed-angle Sorvall rotor. In parallel, a Ni-NTA FF column (Cytiva) was equilibrated with 2 column volumes (CV) of resuspension buffer. The clarified supernatant containing the soluble protein fraction was loaded onto the column. The column was washed with 8 CV of resuspension buffer, followed by low-salt wash buffer (50 mM KCl, 50 mM HEPES pH 7.5, 0.5 mM DTT, 40 mM imidazole). Bound proteins were eluted with elution buffer (50 mM KCl, 50 mM HEPES pH 7.5, 0.5 mM DTT, 500 mM imidazole). The eluate was directly loaded onto a HiTrap Q HP column (Cytiva) equilibrated in low-salt wash buffer. The column was washed with 4 CV of low-salt wash buffer, and proteins were eluted using a linear gradient to 50% high-salt buffer (1 M KCl, 50 mM HEPES pH 7.5, 0.5 mM DTT, 40 mM imidazole) over 10 CV, followed by 2 CV at 100% high-salt buffer. Peak fractions were analyzed by SDS-PAGE, pooled, and concentrated using 30 kDa MWCO centrifugal filters (Amicon). The sample was further purified by size-exclusion chromatography on a Superdex 200 HiLoad 16/600 column (Cytiva) equilibrated in minimal buffer (30 mM KCl, 10 mM HEPES). Fractions containing GADD34Δ240 were identified by SDS–PAGE, pooled, concentrated using the same Amicon centrifugal filters, aliquoted, flash-frozen in liquid nitrogen, and stored at −80 °C until further use.

### In vitro transcription (IVT), RNC generation, and purification

The sequence of human ANXA4 containing a mouse-derived N terminus^38^ was cloned into the pTWIST vector described previously^24^. The PCR-amplified in vitro transcription template contained a T7 RNA polymerase promoter, an EMCV IRES sequence for cap-independent translation initiation, eGFP, a 22xGS linker, a mutated SUMO tag for purification, 36 residues of human ANXA4 with the modified N-terminus, and a mutated Xbp1 arrest peptide to stall the nascent chain, followed by a VV linker and a 6xHis tag to identify failed stalling events (Supplementary Fig. 6).

For IVT, 800 ng of DNA template was transcribed for 2 h at 37 °C using T7 RNA polymerase at the final concentration of 1.15 mg/ml produced in-house. The reaction was carried out in transcription buffer containing 40 mM Tris-HCl pH 7.6, 1 mM DTT, 2 mM spermidine, 24 mM MgCl₂, 0.4 U/µl RNase inhibitor (RNaseOUT), and 5 mM NTPs. Residual DNA template was removed by RNAse-free DNaseI (Qiagen) treatment for 15 min at 37 °C. Excess pyrophosphate generated during transcription was pelleted by centrifugation, and the RNA-containing supernatant was subjected to LiCl precipitation (2.3 M final concentration) for 30 min at −20 °C. RNA was pelleted by centrifugation for 20 min, washed with freshly prepared 70% ethanol, and air-dried. The purified RNA was resuspended in Milli-Q water, analyzed by agarose bleach gel electrophoresis, and quantified by UV absorbance to assess concentration and purity.

To generate Xbp1-stalled RNCs, HeLa cell extract (HCE) (Ipracell, CC-01-40-50) was supplemented as described previously for translation of EMCV IRES-containing mRNAs with minor modifications^54^. HCE was thawed on ice and pre-incubated at 37 °C for 5 min in the presence of GADD34Δ240, added at a concentration that resulted in 7.5 µg/ml in the final translation reaction. GADD34Δ240 mediates dephosphorylation of eIF2, thereby preventing inhibition of global translation and improving both RNC yield and protein expression in an in vitro translation systems^54–56^. A supplement mixture was then added to the extract to generate the human in vitro translation system (HITS). In the final translation reaction, this supplement provided 120 mM KCl, 15 mM HEPES (pH 7.0), 20 mM creatine phosphate, and 0.9 mM MgCl₂, while HCE constituted 75% of the total reaction volume. The supplemented extract was aliquoted, flash-frozen, and stored until use.

Purified mRNA was heated to 65 °C for 5 min, cooled on ice for 1 min, and added to HITS at a 1:5.13 ratio to yield a final mRNA concentration of 100 nM. Translation reactions were incubated at 33 °C for 20 min and subsequently quenched by transferring the samples to ice.

Purification of RNCs was performed as described previously with slight modifications^24^ (detailed protocol for anti-GFP nanobody based purification was described elsewhere^57^). Before batch binding of GFP-tagged in vitro translated stalled RNCs, 180 μL of magnetic streptavidin beads (Pierce) were washed with low salt purification buffer (50 mM HEPES pH 7.5, 100 mM KCl, 5 mM MgCl_2_, and 0.1% (v/v) Triton X-100) and incubated with biotinylated anti-GFP nanobody for 15 minutes at 4 °C with head over-tail-mixing (20 µg of nanobody per 60 µl of beads slurry) and blocked with 100 µM of biotin. The nanobody-decorated streptavidin beads were mixed with stalled RNCs generated earlier, which were diluted two-fold and supplemented with Triton X-100 to 0.1% final concentration. Following batch binding, the magnetic beads were washed twice with low-salt buffer and twice with high-salt buffer (50 mM HEPES pH 7.5, 500 mM KCl, 5 mM MgCl_2_, and 0.1% (v/v) Triton X-100). Finally, beads were washed once more with low-salt buffer. RNCs were eluted by incubating the beads for 20 min with 250 nM SENP^EuB^ protease, with gentle mixing every 5 min. Elution was repeated if necessary until the UV absorbance signal was no longer detected. Eluted fractions were pooled and centrifuged for 3-5 min to remove residual magnetic beads. RNCs were subsequently ultracentrifuged at 153,700 x g for 2 h at 4 °C using a TLA-55 rotor (Beckman Colter). The supernatant was removed, and the pellet was resuspended in reaction assembly buffer (50 mM HEPES pH 7.5, 100 mM KOAc, 5 mM Mg(OAc)_2_). The final RNC concentration was determined using a calculated theoretical extinction coefficient for human 80S including the mRNA sequence. RNCs used in this study were obtained from two independent preparations.

### Complex assembly, sample vitrification, and cryo-EM data collection

To assemble the complex for structural studies, RNCs (70 nM), NAC (500 nM), and NatB (1 µM) were combined at the indicated final concentrations. RNCs were first mixed with NAC and incubated at room temperature for 10 min, followed by the addition of NatB and a further 10 min incubation. The reaction was carried out in reaction assembly buffer in the presence of 1.5 µM coenzyme A and 0.02% C12E8 detergent. The final sample was kept on ice until cryo-EM grid preparation.

Quantifoil R2/2 300 mesh holey carbon copper grids were coated with an additional continuous layer of homemade thin amorphous carbon and plasma cleaned at 15 mA for 15 s using a PELCO easiGlow glow discharge cleaning system (Ted Pella, Inc.). Grids were mounted in a Vitrobot Mark IV (Thermo Fisher Scientific) operating at 8 °C and 100% humidity. A total of 3.5 µl of sample was applied to each grid, incubated for 30 s, and blotted from both sides for 6–8 s with a blot force of 15 using standard Vitrobot filter paper (Ted Pella, 47000-100). Grids were immediately plunge-frozen into a liquid ethane-propane mixture (1:2) for vitrification and stored in liquid nitrogen until data collection.

Cryo-EM images were collected at the ETH Scientific Center for Optical and Electron Microscopy (ScopeM) using a Titan Krios G3i transmission electron microscope (Thermo Fisher Scientific) operating at 300 kV and equipped with a Gatan K3 direct detection camera and a Gatan BioQuantum energy filter. The energy filter slit was set to 20 eV, and the defocus was varied between −0.9 µm and −2.4 μm in 0.3 μm steps. Data were collected at a nominal magnification of 81,000x using automated acquisition with the EPU software (Thermo Fisher Scientific). The total number of 20,701 movies were collected at the calibrated pixel size of 1.049 Å per pixel with a total electron dose of 50 e^−^ Å^−2^ over two independent sessions.

### Cryo-EM data processing

The two datasets were initially processed separately and later merged (see Supplementary Fig. 1). All data processing was performed in CryoSPARC v4.6.2.-v5.0.3^58^. For the first dataset, 10,131 movies were motion-corrected and subjected to contrast transfer function (CTF) parameters estimation. Ribosomal particles were picked using blob picking with a particle diameter range of 250–350 Å and extracted using a box size of 560 pixels, which was subsequently Fourier-cropped to 180 pixels, resulting in a final pixel size of 3.3 Å per pixel. Extracted particles were subjected to 2D classification using a 400 Å circular mask with 200 classes. Selected particles corresponding to ribosome-like classes were subjected to homogeneous refinement and retained for merging with the second dataset.

For the second dataset, 10,570 movies were motion corrected, CTF parameters were estimated, and particles were picked and extracted in CryoSPARC Live using the same parameters as described above. Extracted particles were first subjected to ab-initio reconstruction without alignments into four classes to generate noisy decoy volumes. In parallel, 2D classification was performed on all particles, and a subset of ribosome-like classes was selected and used for single-class ab-initio reconstruction. The four decoy and the ribosomal ab-initio volumes were then used as references for three rounds of heterogeneous refinement. Particles corresponding to intact 80S ribosomes were selected and subjected to homogeneous refinement.

Particles from both datasets were then merged and refined together. The per-particle scale factors were used to identify particle subsets clustered by Gaussian fitting, allowing removal of lower-quality particles. The higher-quality particle set was subjected to homogeneous refinement and further analyzed by 3D variability analysis (3DVA) using five modes and a filter resolution of 10 Å, which enabled removal of particles corresponding to isolated 60S subunits. The remaining 80S ribosomes were then classified according to their translational state using a circular mask covering the decoding center. For this, 3D variability analysis with a filter resolution of 8 Å and a single mode was used to separate particles into distinct classes corresponding to P-site tRNA RNCs and collided ribosomes containing A/P-and E-site tRNAs. Subsequently, additional 3D variability analysis was performed on P-site tRNA RNCs using a mask positioned near the peptide exit tunnel, which allowed efficient classification of particles based on the presence or absence of NatB. Particles corresponding to NatB-bound P-site tRNA RNCs were re-extracted at the full pixel size of 1.049 Å per pixel. The resulting particles were subjected to a series of homogeneous refinements, with optimization of per-particle scale, per-group CTF parameters, and per-particle defocus. The resulting reconstruction was subjected to a final round of global classification into two classes using a 6 Å low-pass filter to assess potential compositional variability within the dataset. This procedure yielded the final map comprising 63,788 particles at a global resolution of 3.19 Å, showing well-resolved density for NAC and NatB positioned near the peptidyl-tunnel exit. All resulting maps generated during data processing were visually inspected using UCSF Chimera v1.17.2 or UCSF ChimeraX v1.10.1^59^.

### Model building and refinement

As a starting model, the structure of the 80S human ribosome harboring a XBP1u-stalled nascent chain and NAC^17^ was docked into the cryo-EM map using the UCSF ChimeraX v1.10.1 software (PDB ID: 28LN)^59–61^. An AlphaFold-predicted model of the NatB-NAC heterotetramer was rigid-body fitted into the cryo-EM map after removal of the NAC globular domain, leaving only the C-terminal UBA domain of NACα. The nascent chain residues were altered to match the sequence of generated RNCs. The model was adjusted manually in Coot 0.9.8.92^62^ to obtain a better fit of the model to the cryo-EM density map. The updated model was fully assembled in PyMOL v3.1.6.1 and subjected to real-space coordinate and ADP refinement in Phenix 2.0_5936 using Ramachandran restrains^63,64^. The final model geometry was validated using MolProbity 4.5.2^65^ (Supplementary Table 1), while the quality of the model-to-map fit was indicated by a high CC_mask_ = 0.84 value and by comparing the model vs. map FSC at a value of 0.5 (3.3 Å, masked), which coincided well with the resolution of the map obtained using the FSC = 0.143 criterion between map half-sets (3.19 Å). The final structure was visualized using ChimeraX^59^, and the resulting images were used to prepare the figures in the manuscript.

### In vitro ribosome co-sedimentation assay

Human ribosomes were purified from HEK293T cells as previously described^15^. Purified NatB and NAC variants were incubated with ribosomes (250 nM ribosomes, 40 nM NatB, 500 nM NAC) for 10 min at 12 °C in ribosome sedimentation buffer (30 mM HEPES pH 7.4, 100 mM potassium acetate, 5 mM MgCl_2_, 2 mM DTT, and 1.5x protease inhibitor cocktail [Roche]). Samples were layered onto a 25% sucrose cushion prepared in the same buffer and subjected to ultracentrifugation at 220,000 x g for 90 min at 4 °C. Ribosomal pellets and total input fractions were analyzed by SDS-PAGE followed by immunoblotting. NatB competition assays with other N-acetyltransferases (NatA and NatD) were performed under similar conditions. At least three independent biological replicates of in vitro ribosome co-sedimentation experiments were performed.

### Human cell culture and transfection

HEK293T cells (ATCC, CRL-3216) were maintained in Dulbecco’s modified Eagle’s medium (DMEM) supplemented with 10% fetal calf serum and Normocin (100 µg/ml) at 37 °C in a humidified 5% CO_2_ atmosphere. Genetic manipulations were performed as described previously^20,22,24^. Briefly, cells were transfected with plasmid DNA and siRNA by electroporation in Opti-MEM using a NEPA21 electroporator (Nepagene). For knockdown experiments, cells were transfected with 2 µg siRNA duplexes (Biomers) targeting the 3′ untranslated regions (3′UTRs) of NACα (5′-AGGAGUAACUGCAGCUUGG-dTdT-3′), NAA25 (5′-GCAUAUGGCAGCAACAAGA-dTdT-3′), or NAA20 (5′-GUUCUUAGGCAGAUACUCU-dTdT-3′ and 5′-GGAUGAUUCUGGAGCUCUA-dTdT-3′). Control cells received a nonspecific (ns) siRNA (5′-UUCUCCGAACGUGUCACGU-dTdT-3′). Where indicated, depleted proteins were re-expressed by co-transfection of plasmids encoding the corresponding cDNAs under the control of a CMV promoter and an SV40 3′UTR. NACα and NAA25 variants were expressed with an N-terminal 3xFLAG tag, whereas NAA20 variants were untagged.

### Ribosome binding assay in cells

Ribosome co-sedimentation assays were performed as previously described^20,24^. Cells were harvested 48 h after transfection and lysed in ice-cold buffer (30 mM HEPES pH 7.4, 100 mM potassium acetate, 5 mM MgCl_2_, 5% mannitol, 0.04% octaethylene glycol monododecyl ether, 100 µg/ml cycloheximide, 1 mM DTT, and 1x protease inhibitor cocktail [Roche]). Lysates were cleared by centrifugation and layered onto a 25% sucrose cushion prepared in the same buffer and centrifuged at 220,000 x g for 90 min at 4 °C. Ribosomal pellets and total input fractions were analyzed by SDS-PAGE and immunoblotting. At least three independent biological replicates of ribosome co-sedimentation experiments were performed.

### Analysis of NatB-dependent Nt-acetylation in cells

NatB activity in cells was assessed by co-transfection of a pEF-BOS vector encoding mouse ANXA4 (amino acids 1-14) fused to GFP-FLAG (2 µg). Cells were lysed in reporter lysis buffer (50 mM Tris-HCl pH 7.5, 150 mM NaCl, 2 mM EDTA, 1 mM EGTA, 0.5% NP-40, and 1x protease inhibitor cocktail [Roche]) and cleared by two rounds of centrifugation (13,000 x g, 5 min, 4 °C). Proteins were analyzed by SDS-PAGE and immunoblotting. Nt-acetylated ANXA4 was detected using an Nt-acetyl-specific anti-ANXA4 antibody (B4-IgM^38^), and total reporter levels were detected using anti-FLAG or anti-GFP antibodies (see Antibodies section). At least three independent biological replicates of Nt-acetylation experiments were performed.

### Antibodies

The following antibodies were used for immunoblotting: anti-ANXA4 (clone B4-IgM) (Liudmila Kulik, University of Colorado Denver, 1:2000 dilution); anti-FLAG (clone M2) (Merck, F1804, 1:5000 dilution); anti-FLAG (polyclonal) (Merck, F7425, 1:2000 dilution); anti-GFP (clone 7.1/13.1) (Merck, 11814460001, 1:1000 dilution); anti-NAA10 (clone A-10) (Santa Cruz, sc-373920, 1:1000 dilution); anti-NAA15 (clone D-7) (Santa Cruz, sc-365931, 1:1000 dilution); anti-NAA20 (clone 36-8) (Santa Cruz, sc-100645, 1:1000 dilution); anti-NAA20 (polyclonal) (Proteintech, 15807-1-AP, 1:2000 dilution); anti-NAA25 (polyclonal) (Invitrogen, PA5-97099, 1:2000 dilution); anti-NAA40 (polyclonal) (Merck, SAB3500167, 1:1000 dilution); anti-NACα (polyclonal) (Biorbyt, orb411671, 1:2000 dilution); anti-NACα (polyclonal) (Proteintech, 32235-1-AP, 1:5000 dilution); anti-NACβ (clone EPR16495) (Abcam, ab203517, 1:2000 dilution); anti-eL19 (clone K-12) (Santa Cruz, sc-100830, 1:1000 dilution); anti-uL22 (polyclonal) (Proteintech, 14121-1-AP, 1:2000 dilution); anti-uL4 (clone RQ-7) (Santa Cruz, sc-100838, 1:2000 dilution).

### Reporting summary

Further information on research design is available in the Nature Portfolio Reporting Summary linked to this article.

## Supplementary information

Supplementary Information

Reporting Summary

Transparent Peer Review file

## Source data

Source Data

## Data Availability

Models and electron microscopy maps were deposited at the PDB and EMDB with accession codes 30HI/000030HI and EMD-57771, respectively. All other data generated or analyzed during this study are available within the Article and Supplementary Files. Reagents required to repeat the experiments reported in this paper are available from the lead contact (E.D.) upon request. Source data are provided with this paper.
