# Supplementary material for: Structural basis of cotranslational protein N-terminal acetylation by NatB in human cells
Source: Nat Commun. 2026 Jul 11;17:6088. doi: 10.1038/s41467-026-75207-1 (PMC13356037; doi:10.1038/s41467-026-75207-1)
Supplement: Supplementary file 1 — Supplementary Information [file 41467_2026_75207_MOESM1_ESM.pdf]

Supplementary Information for

Structural basis of cotranslational protein N-terminal acetylation by NatB in human cells

Natalia Silva Alves[1], Pawel Knejski[2], Alain Scaiola[2], Marc Leibundgut[2], Martin Gamerdinger[1]*, Nenad Ban[2]*, Elke Deuerling[1]*

*corresponding authors:

martin.gamerdinger@uni-konstanz.de

ban@mol.biol.ethz.ch

elke.deuerling@uni-konstanz.de

The PDF file includes:

Supplementary Figures 1-6

Supplementary Table 1

Supplementary References

Supplementary Figure 1: Cryo-EM workflow for RNC^{ANXA4} · NAC · NatB complex

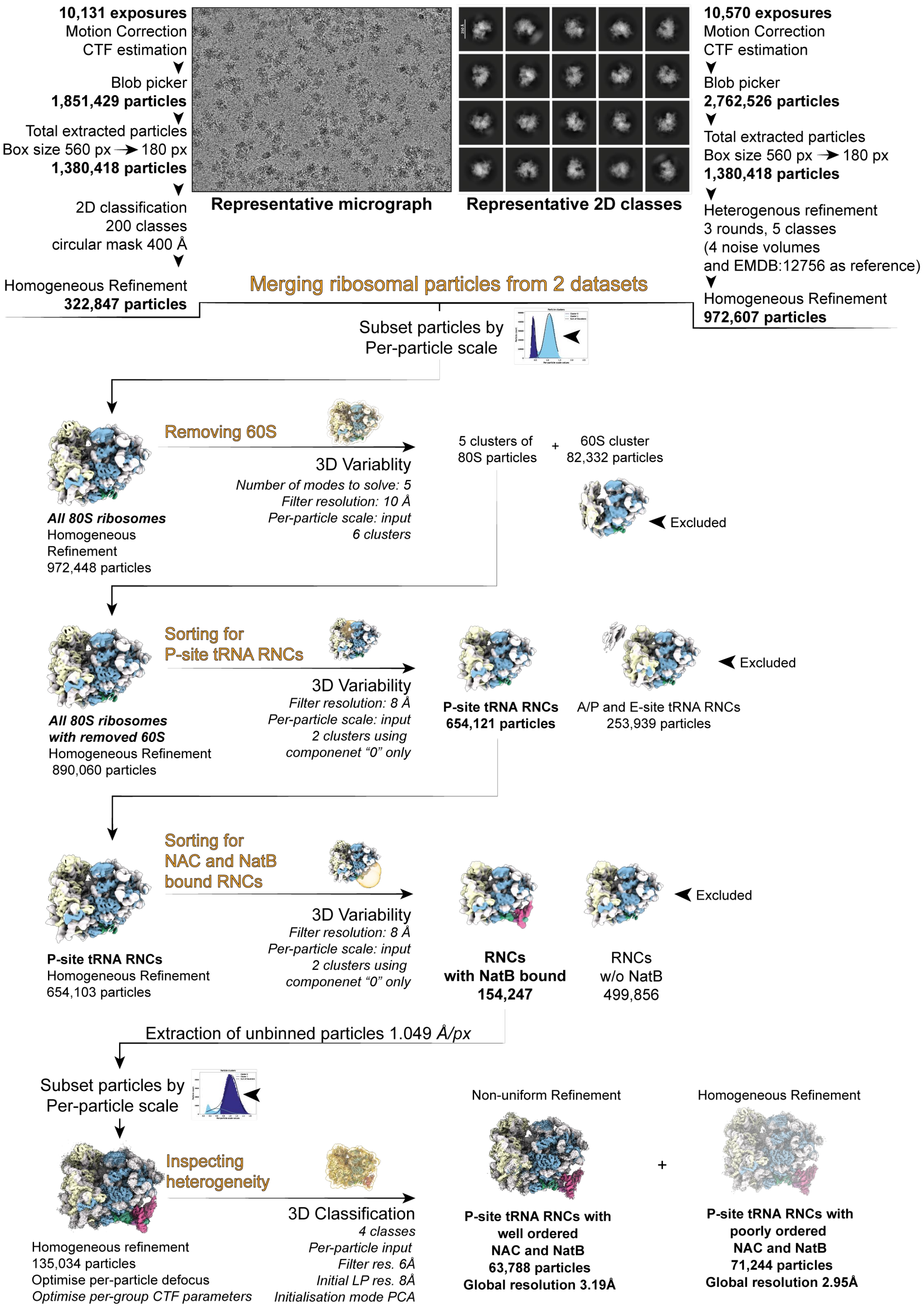

Cryo-EM processing scheme showing the steps to obtain a final high-resolution volume of NAC- and NatB-associated RNCs. All steps were performed using the cryoSPARC software. Numbers of

movies, particles or types of obtained volumes are written in bold, non-default parameters are indicated in italic. Obtained 3D- refined or classified volumes are shown indicating rRNA colored in white, small subunit proteins in yellow, large subunit proteins in blue, the NAC heterodimer in green, NatB in pink. Masks are shown as semi-transparent orange surfaces. Briefly, imported movies originating from 2 datasets were preprocessed independently, and blob picked particles were extracted with a pixel size of 3.3 Å per pixel. Depending on the dataset, ribosomal particles were obtained either from 2D classification or 3D heterogeneous refinement and pooled together for homogeneous refinement. In the initial processing steps, particles were separated based on per-particle scale factors, and low-quality ribosomes were removed. Remaining 80S ribosomes were subjected to 3D variability analysis to eliminate 60S contaminants. Subsequently, ribosomal particles were subjected to two rounds of 3D variability analysis using solvent masks that either cover the decoding center or the peripheral peptidyl tunnel exit, respectively. The obtained NAC-NatB-bound P-site tRNA-stalled RNC particles were extracted with pixel size of 1.049 Å/px and 3D refined to obtain a cryo-EM map with high resolution features. The map was further classified and the volume with well-ordered NAC and NatB was resolved to 3.19 Å global resolution.

Supplementary Figure 2: Local resolution estimation, orientation distribution and validation plot.

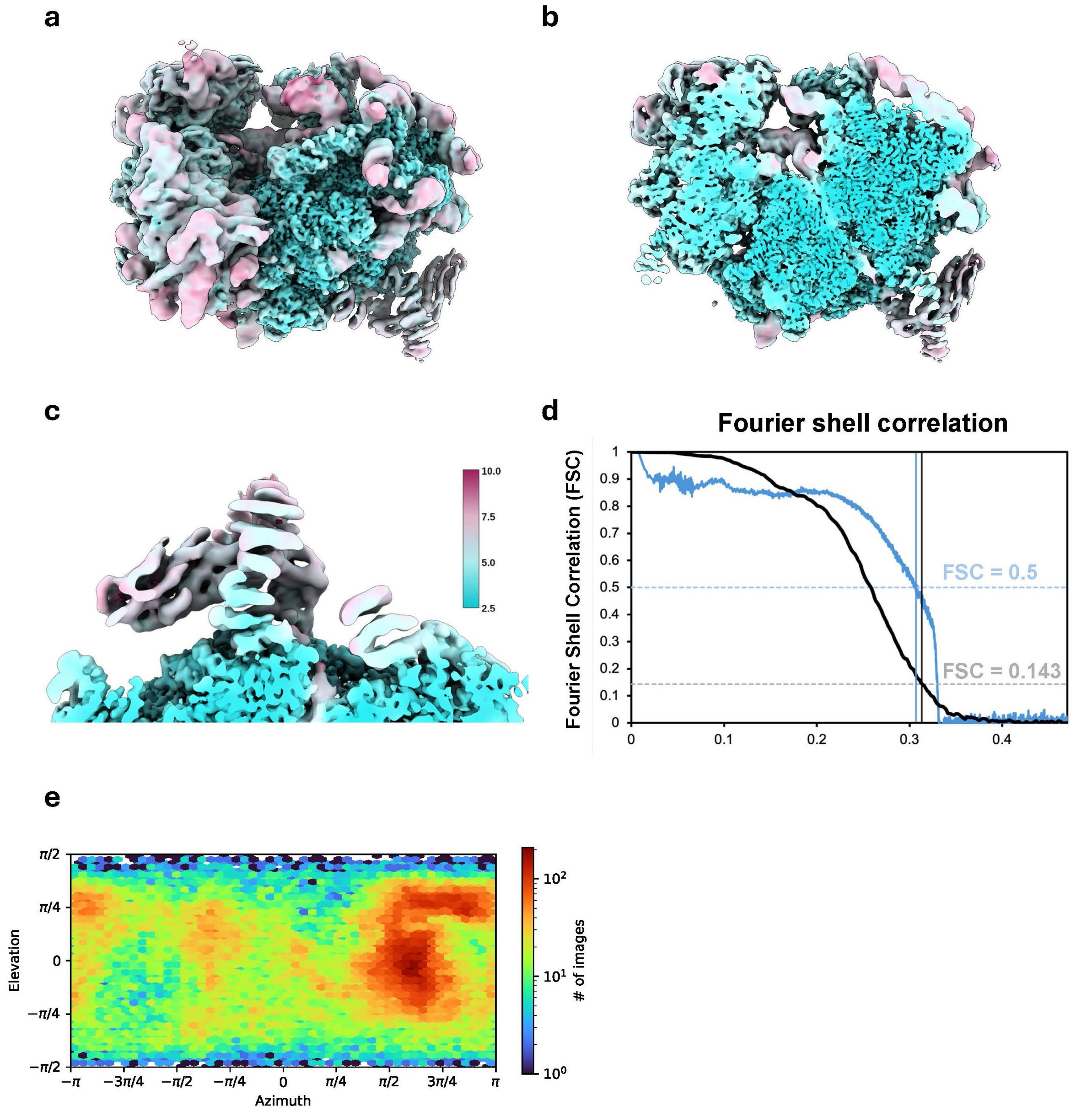

(a) Cryo-EM density map filtered and colored according to local resolution. **(b)** Cross-section of the cryo-EM map shown in (a) to visualize the ribosomal core. **(c)** Close-up view of the NAC-NatB complex shown in (b), with the scale bar shown on the right. **(d)** Fourier shell correlation (FSC) curves showing global resolution estimates. The blue curve represents the model-to-map FSC used for model validation (FSC = 0.5 criterion), and the black curve the map-to-map FSC between half-maps used to determine the overall map resolution (FSC = 0.143 criterion). FSC thresholds are indicated by dashed lines. The proximity of the value between map-versus-map and map-versus-model FSCs indicates the absence of overfitting during refinements **(e)** Angular distribution of the particles shown for the final 3D refinement.

Supplementary Figure 3: Conservation of NatB ribosome contacts.

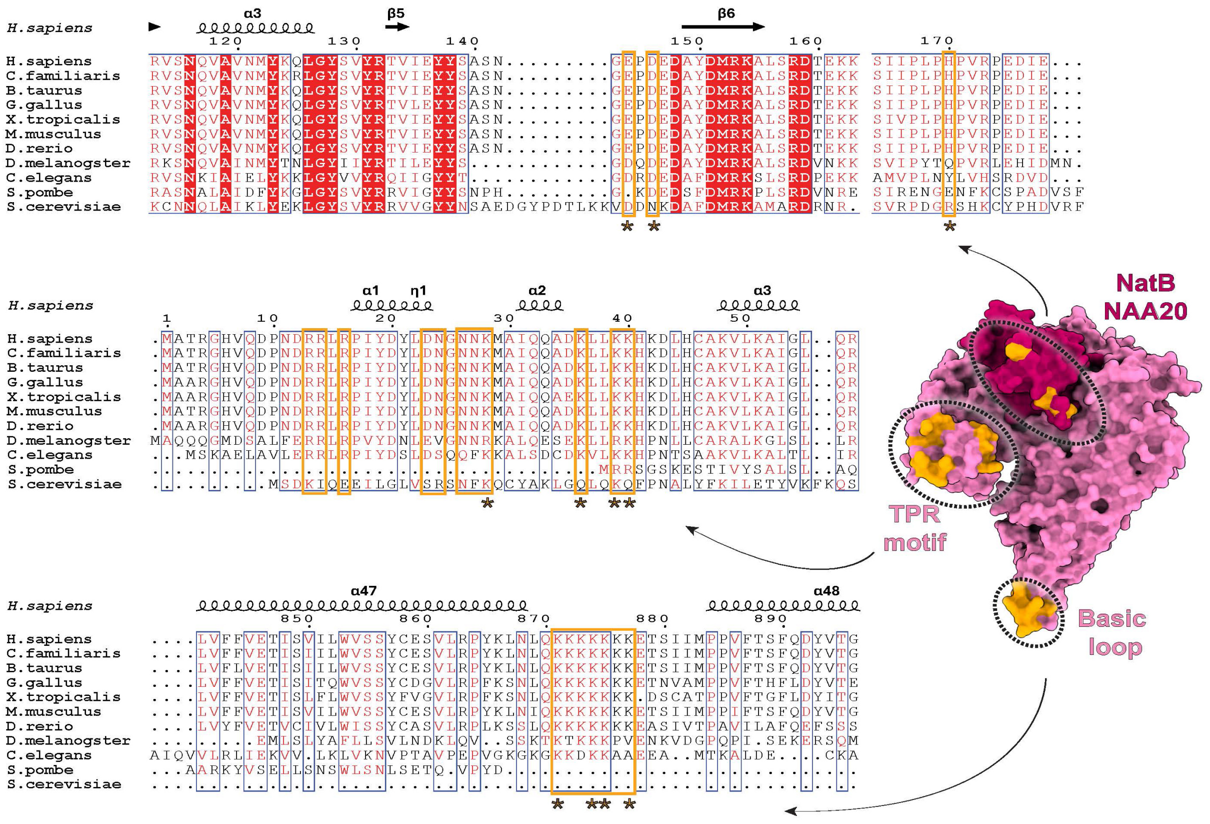

Sequence alignment of NAA25 (pink) and NAA20 (deep magenta) with ribosomal contact points highlighted in orange. Sequences from eight metazoan species and two yeast species were obtained from UniProt and aligned using the Clustal Omega tool[1]. ESPript 3.2[2] was used to visualize the conserved residues, which are shown in red and outlined in blue. Strictly conserved residues are displayed as white text on a red background. Black font is used when no conservation is observed. Amino acids marked with asterisks were mutated in this study.

Supplementary Figure 4: Ribosome binding competition of NatB with NatA and NatD.

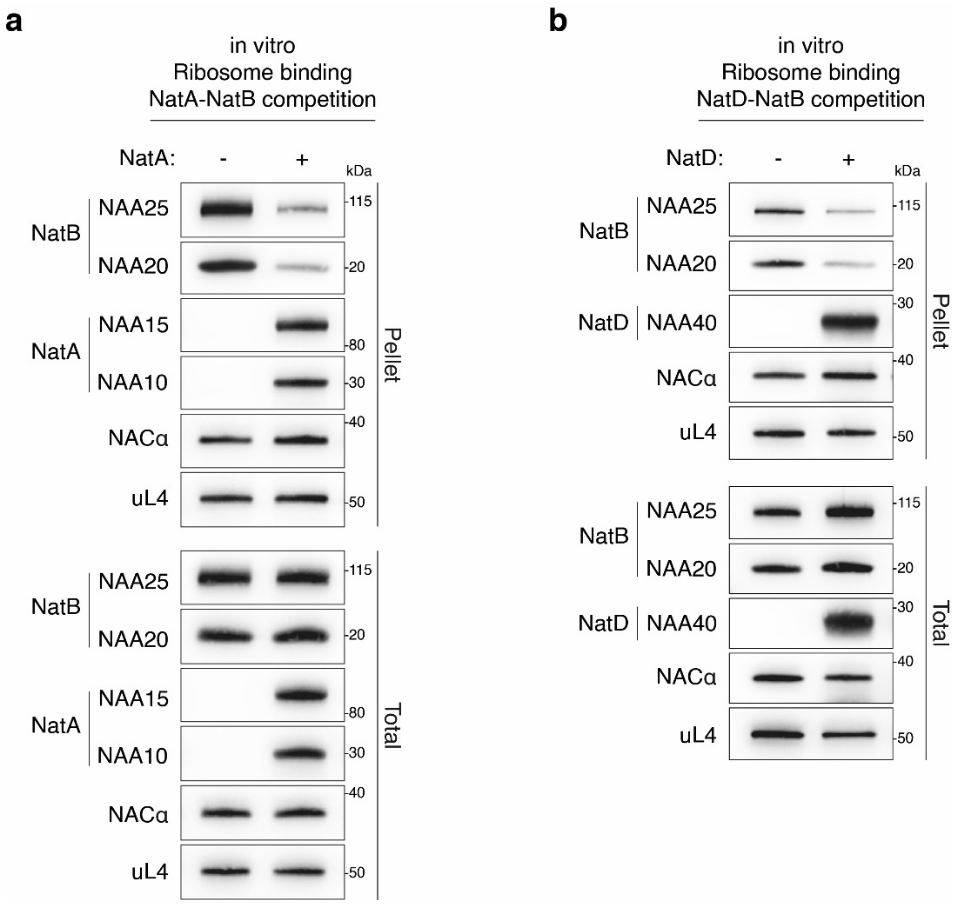

(a) *In vitro* co-sedimentation of purified human NatB with ribosomes in the presence and absence of NatA (NAA15-NAA10 complex). Ribosomal pellet and total fractions were analyzed by immunoblotting. **(b)** *In vitro* co-sedimentation of purified human NatB with ribosomes in the presence and absence of NatD (NAA40). Ribosomal pellet and total fractions were analyzed by immunoblotting.

Supplementary Figure 5: Purity of recombinant protein complexes generated in this study.

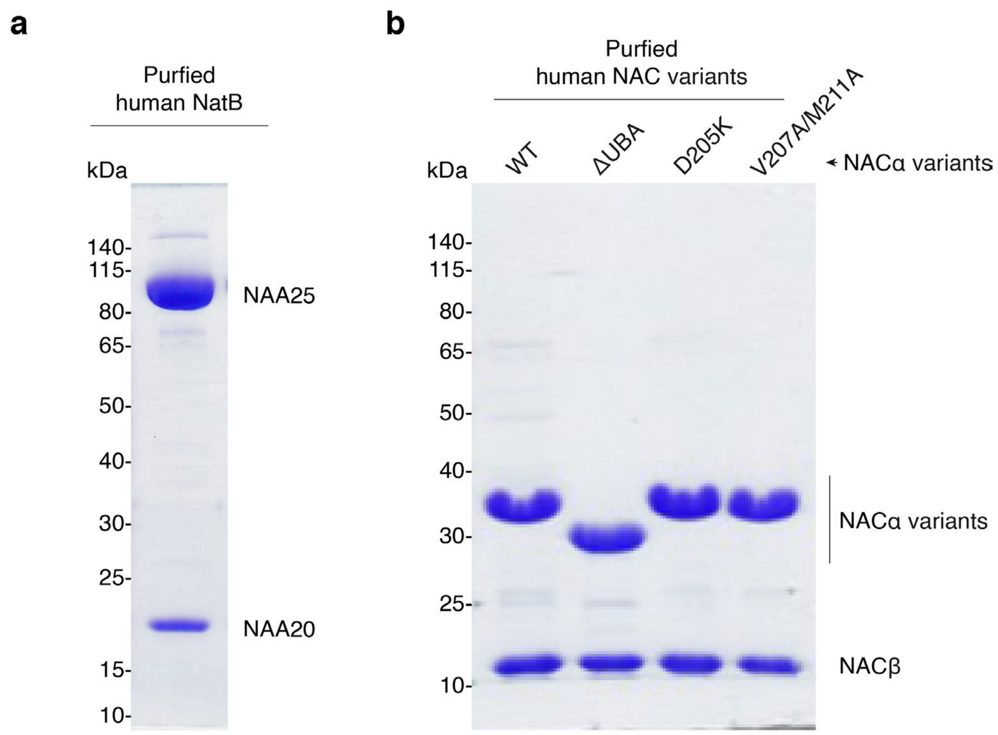

(a) Purified recombinant human NatB complex (NAA25-NAA20). Coomassie stained bis-TRIS PAGE gel is shown. **(b)** Purified recombinant human NAC complexes (NACα-NACβ). NAC variants consisting of WT-NACβ and indicated NACα variants were used in Fig. 4b. Coomassie stained SDS-PAGE gel is shown.

Supplementary Figure 6: Schematic representation of the mRNA architecture.

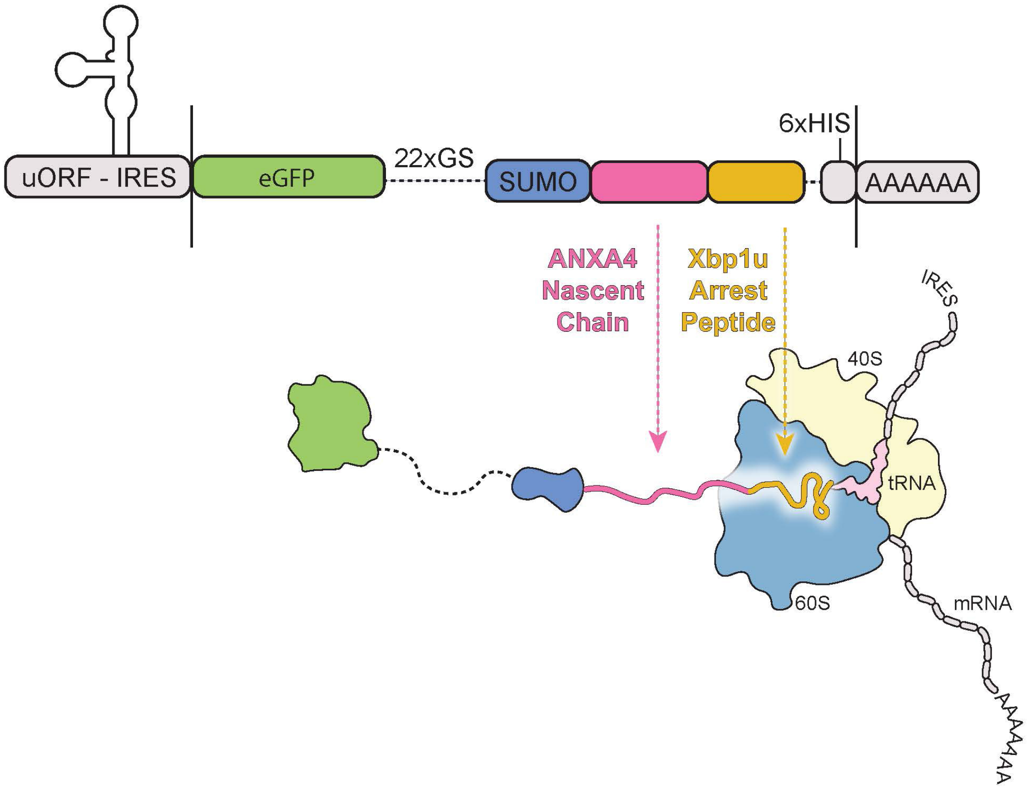

Schematic overview of the mRNA construct highlighting all key elements. The 5' untranslated region contains an EMCV IRES to enable HITS cap-independent translation. The open reading frame begins with eGFP followed by a mutated SUMO tag, separated by a linker, which facilitates purification of RNCs. This is followed by a 36-residue nascent chain derived from mouse ANXA4 and a 24-residue mutated Xbp1u arrest peptide to promote efficient ribosome stalling. The coding sequence terminates with a VV linker and a C-terminal hexahistidine tag, allowing detection of failed stalling events. The 3' end of the mRNA includes a poly(A) tail introduced by PCR prior to *in vitro* transcription. After translation and isolation of RNCs, SUMO cleavage using SENP^{EuB} protease generates a native ANAXA4 N terminus starting with methionine.

Supplementary Table 1: Cryo-EM data collection, refinement and validation statistics

**Complex**	**# Human XBP1u-stalled RNC:NAC:NatB**
EMDB code	EMD-57771
PDB ID / Extended PDB ID	PDB 30HI / pdb_000030HI
**Data collection and processing**	
Microscope	TFS Titan Krios G3i
Camera	Gatan K3
Magnification	81,000 x (nominal)
Voltage (kV)	300
Electron exposure (e–/Å^2)	50
Defocus range (µm)	(-0.9) – (-2.4)
Pixel size (Å)	1.049 (super-resolution pixel at 0.5245Å/pixel)
Symmetry imposed	C1
Automation software	EPU
Energy filter slit width	20 eV
Micrographs collected	20,701
Micrographs used	20,701
Initial particle images (no.)	2,739,042
Final particle images (no.)	63,788
Map resolution (Å)	3.19
FSC threshold	0.143
Map resolution range (Å)	2.2 – 10
Map sharpening *B* factor (Å^2)	43.3
**Refinement**	
Initial model used (PDB code)	28LN
Model resolution (Å)	3.3
FSC threshold	0.5
CCmask	0.84
Model composition	
Non-hydrogen atoms	232,781
Protein residues	13,369
RNA residues	5,819
Ligands Mg^{2+}/Zn^{2+}/CoA	453/8/1
B factors min/max/mean (Å^2)	
Protein	43.71/578.89/181.51
RNA	44.98/948.09/201.62
Ligand	40.34/417.05/124.34
**Validation**	
General	
R.m.s. deviations	
Bond lengths (Å)	0.003
Bond angles (°)	0.75
MolProbity score	1.23
Clashscore	3.35
Protein	
Poor rotamers (%)	1.4
CaBLAM outliers (%)	1.2
EM Ringer score	2.1
Ramachandran plot	
Favored (%)	98.26
Allowed (%)	1.72
Disallowed (%)	0.02
RNA	
Pucker outliers (%)	0.4
Bond outliers (%)	0.2
Angle outliers (%)	0
Suite outliers (%)	14.8

Supplementary References

1. Madeira F*, et al*. The EMBL-EBI Job Dispatcher sequence analysis tools framework in 2024. *Nucleic Acids Res* **52**, W521-W525 (2024).

2. Robert X, Guillon C, Gouet P. FoldScript: a web server for the efficient analysis of AI-generated 3D protein models. *Nucleic Acids Res* **53**, W277-W282 (2025).
