# Supplementary material for: Structural basis of cotranslational protein N-terminal acetylation by NatB in human cells
Source: Nat Commun. 2026 Jul 11;17:6088. doi: 10.1038/s41467-026-75207-1 (PMC13356037; doi:10.1038/s41467-026-75207-1)
Supplement: Supplementary file 2 — Reporting Summary [file 41467_2026_75207_MOESM2_ESM.pdf]

nature portfolio

Corresponding author(s): Martin Gamerdinger, Nnead Ban, Elke Deuerling

Last updated by author(s): Jun 9, 2026

Reporting Summary

Nature Portfolio wishes to improve the reproducibility of the work that we publish. This form provides structure for consistency and transparency in reporting. For further information on Nature Portfolio policies, see our Editorial Policies and the Editorial Policy Checklist.

Statistics

For all statistical analyses, confirm that the following items are present in the figure legend, table legend, main text, or Methods section.

n/a	Confirmed	
☐	☒	The exact sample size (*n*) for each experimental group/condition, given as a discrete number and unit of measurement
☒	☐	A statement on whether measurements were taken from distinct samples or whether the same sample was measured repeatedly
☒	☐	The statistical test(s) used AND whether they are one- or two-sided<br>*Only common tests should be described solely by name; describe more complex techniques in the Methods section.*
☒	☐	A description of all covariates tested
☒	☐	A description of any assumptions or corrections, such as tests of normality and adjustment for multiple comparisons
☐	☒	A full description of the statistical parameters including central tendency (e.g. means) or other basic estimates (e.g. regression coefficient) AND variation (e.g. standard deviation) or associated estimates of uncertainty (e.g. confidence intervals)
☒	☐	For null hypothesis testing, the test statistic (e.g. *F*, *t*, *r*) with confidence intervals, effect sizes, degrees of freedom and *P* value noted<br>*Give P values as exact values whenever suitable.*
☒	☐	For Bayesian analysis, information on the choice of priors and Markov chain Monte Carlo settings
☒	☐	For hierarchical and complex designs, identification of the appropriate level for tests and full reporting of outcomes
☒	☐	Estimates of effect sizes (e.g. Cohen's *d*, Pearson's *r*), indicating how they were calculated

Our web collection on statistics for biologists contains articles on many of the points above.

Software and code

Policy information about availability of computer code

Data collection	EPU 3.10.0.8733 (ThermoFisher)
Data analysis	CryoSPARC v4.6.2-v5.0.3;<br>Coot 0.9.8.92;<br>Phenix v2.0-5936;<br>PYMOL v3.1.6.1;<br>UCSF ChimeraX v1.10.1;<br>UCSF Chimera v1.17.2;<br>MOL Probity 4.5.2

For manuscripts utilizing custom algorithms or software that are central to the research but not yet described in published literature, software must be made available to editors and reviewers. We strongly encourage code deposition in a community repository (e.g. GitHub). See the Nature Portfolio guidelines for submitting code & software for further information.

Data

Policy information about availability of data

All manuscripts must include a data availability statement. This statement should provide the following information, where applicable:

- Accession codes, unique identifiers, or web links for publicly available datasets
- A description of any restrictions on data availability
- For clinical datasets or third party data, please ensure that the statement adheres to our policy

Models and electron microscopy maps were deposited at the PDB and EMDB with accession codes 30HI [http://doi.org/10.2210/pdb_000030hi/pdb] and EMD-57771 [https://www.ebi.ac.uk/emdb/EMD-57771], respectively. All other data generated or analyzed during this study are available within the Article and Supplementary Files. Reagents required to repeat the experiments reported in this paper are available from the lead contact (E.D.) upon request. Source data are provided with this paper.

Research involving human participants, their data, or biological material

Policy information about studies with human participants or human data. See also policy information about sex, gender (identity/presentation), and sexual orientation and race, ethnicity and racism.

Reporting on sex and gender	n/a
Reporting on race, ethnicity, or other socially relevant groupings	n/a
Population characteristics	n/a
Recruitment	n/a
Ethics oversight	n/a

Note that full information on the approval of the study protocol must also be provided in the manuscript.

Field-specific reporting

Please select the one below that is the best fit for your research. If you are not sure, read the appropriate sections before making your selection.

☒ Life sciences ☐ Behavioural & social sciences ☐ Ecological, evolutionary & environmental sciences

For a reference copy of the document with all sections, see nature.com/documents/nr-reporting-summary-flat.pdf

Life sciences study design

All studies must disclose on these points even when the disclosure is negative.

Sample size	No statistical method was used to predetermine sample size. Sample sizes were chosen based on established practice in the fields of structural biology, biochemistry, and cell biology, and were comparable to those used in previous studies of ribosome-associated protein biogenesis factors. All key findings were reproduced in independent biological replicates, and the sample sizes were sufficient to provide robust and reproducible results.
Data exclusions	No data are excluded from the analysis.
Replication	Cell and in vitro biochemical experiments were performed at least three times as independent biological replicates. The number of biological replicates for each experiment is indicated in the figure legends.
Randomization	Randomization was not applicable because the study did not involve allocation of subjects or samples to treatment groups. Instead, experiments consisted of predefined biochemical, structural, and genetic comparisons designed to test specific hypotheses, with samples analyzed using standardized procedures and objective readouts.
Blinding	Blinding was not relevant to this study because all analyses were based on objective structural, biochemical, and quantitative measurements. Cryo-EM reconstructions were generated through standardized image-processing pipelines, and biochemical and cell assays were evaluated using predefined quantitative readouts. No subjective scoring of experimental outcomes was performed.

Reporting for specific materials, systems and methods

We require information from authors about some types of materials, experimental systems and methods used in many studies. Here, indicate whether each material, system or method listed is relevant to your study. If you are not sure if a list item applies to your research, read the appropriate section before selecting a response.

Materials & experimental systems

n/a	Involved in the study
☐	☒ Antibodies
☐	☒ Eukaryotic cell lines
☒	☐ Palaeontology and archaeology
☒	☐ Animals and other organisms
☒	☐ Clinical data
☒	☐ Dual use research of concern
☒	☐ Plants

Methods

n/a	Involved in the study
☒	☐ ChIP-seq
☒	☐ Flow cytometry
☒	☐ MRI-based neuroimaging

Antibodies

Antibodies used

anti-ANXA4 (clone B4-IgM) (Liudmila Kulik, University of Colorado Denver, 1:2000 dilution); anti-FLAG (clone M2) (Merck, F1804, 1:5000 dilution); anti-FLAG (polyclonal) (Merck, F7425, 1:2000 dilution); anti-GFP (clone 7.1/13.1) (Merck, 11814460001, 1:1000 dilution); anti-NAA10 (clone A-10) (Santa Cruz, sc-373920, 1:1000 dilution); anti-NAA15 (clone D-7) (Santa Cruz, sc-365931, 1:1000 dilution); anti-NAA20 (clone 36-8) (Santa Cruz, sc-100645, 1:1000 dilution); anti-NAA20 (polyclonal) (Proteintech, 15807-1-AP, 1:2000 dilution); anti-NAA25 (polyclonal) (Invitrogen, PA5-97099, 1:2000 dilution); anti-NAA40 (polyclonal) (Merck, SAB3500167, 1:1000 dilution); anti-NACα (polyclonal) (Biorbyt, orb411671, 1:2000 dilution); anti-NACα (polyclonal) (Proteintech, 32235-1-AP, 1:5000 dilution); anti-NACβ (clone EPR16495) (Abcam, ab203517, 1:2000 dilution); anti-eL19 (clone K-12) (Santa Cruz, sc-100830, 1:1000 dilution); anti-uL22 (polyclonal) (Proteintech, 14121-1-AP, 1:2000 dilution); anti-uL4 (clone RQ-7) (Santa Cruz, sc-100838, 1:2000 dilution).

Validation

anti-ANXA4 (clone B4-IgM) (1:2000 dilution) - validated by Kulik et al. Mol Immunol 157, 112-128 (2023) against Nt-acetyl mouse ANXA4.
anti-FLAG (clone M2) (1:5000 dilution) - FLAG epitope antibody, species-independent. Validated by manufacturer (https://www.sigmaaldrich.com/DE/de/product/sigma/b3111).
anti-FLAG (polyclonal) (1:2000 dilution) - FLAG epitope antibody, species-independent.Validated by manufacturer (https://www.sigmaaldrich.com/DE/de/product/sigma/f7425).
anti-GFP (clone 7.1/13.1) (1:1000 dilution) - GFP fusion antibody, species-independent. Validated by manufacturer (https://www.sigmaaldrich.com/DE/de/product/roche/11814460001).
anti-NAA10 (clone A-10) (1:1000 dilution) - validated by manufacturer for immunoblotting using several human cell lines (e.g. Jurkat, https://www.scbt.com/p/ard1-antibody-a-10).
anti-NAA15 (clone D-7) (1:1000 dilution) - validated by manufacturer for immunoblotting using several human cell lines (e.g. HeLa, https://www.scbt.com/p/narg1-antibody-d-7).
anti-NAA20 (clone 36-8) (1:1000 dilution) - validated by manufacturer for immunoblotting using several human cell lines (e.g. HEK293T, https://www.scbt.com/de/p/nat-5-antibody-36-8).
anti-NAA20 (polyclonal) (1:2000 dilution) - validated by manufacturer for immunoblotting using several human cell lines (e.g. HEK293T, https://www.ptglab.com/products/NAT5-Antibody-15807-1-AP.htm).
anti-NAA25 (polyclonal) (1:2000 dilution) - validated by manufacturer for immunoblotting using mouse/rat tissue (https://www.thermofisher.com/antibody/product/C12orf30-Antibody-Polyclonal/PA5-97099).
anti-NAA40 (polyclonal) (1:1000 dilution) - validated by manufacturer for immunoblotting using human thymus tissue (https://www.sigmaaldrich.com/DE/de/product/sigma/sab3500167).
anti-NACα (polyclonal - Biorbyt) (1:2000 dilution) - validated by manufacturer for immunoblotting using several human cell lines (e.g. HeLa, https://www.biorbyt.com/naca1-polyclonal-antibody-orb669944.html).
anti-NACα (polyclonal - Proteintech) (1:5000 dilution) - validated by manufacturer for immunoblotting using several human cell lines (e.g. HeLa, https://www.ptglab.com/products/NACA-Antibody-32235-1-AP.htm).
anti-NACβ (clone EPR16495) (1:2000 dilution) - validated by manufacturer for immunoblotting using several human cell lines (e.g. HeLa, https://www.abcam.com/en-us/products/primary-antibodies/btf3-antibody-epr16495-ab203517).
anti-eL19 (clone K-12) (1:1000 dilution) - validated by manufacturer for immunoblotting using several human cell lines (e.g. HeLa, https://www.scbt.com/de/p/ribosomal-protein-l19-antibody-k-12).
anti-uL22 (polyclonal) (1:2000 dilution) - validated by manufacturer for immunoblotting using several human cell lines (e.g. HeLa, https://www.ptglab.com/de/products/RPL17-Antibody-14121-1-AP.htm).
anti-uL4 (clone RQ-7) (1:2000 dilution) - validated by manufacturer for immunoblotting using several human cell lines (e.g. HeLa, https://www.scbt.com/de/p/ribosomal-protein-l4-antibody-rq-7).

Eukaryotic cell lines

Policy information about cell lines and Sex and Gender in Research

Cell line source(s)	HEK293T cells (RRID: CVCL_0063) were from ATCC (#CRL-3216).
Authentication	Cell line was not authenticated.
Mycoplasma contamination	Cell lines were negative for mycoplasma.

Commonly misidentified lines (See ICLAC register)	None used

Plants

Seed stocks	n/a
Novel plant genotypes	n/a
Authentication	n/a
