# Supplementary material for: Structural basis of cotranslational protein N-terminal acetylation by NatB in human cells
Source: Nat Commun. 2026 Jul 11;17:6088. doi: 10.1038/s41467-026-75207-1 (PMC13356037; doi:10.1038/s41467-026-75207-1)
Supplement: Supplementary file 3 — Transparent Peer Review file [file 41467_2026_75207_MOESM3_ESM.pdf]

nature portfolio

Peer Review File

Structural basis of cotranslational protein N-terminal acetylation by NatB in human cells

Corresponding Author: Professor Elke Deuerling

This file contains all reviewer reports in order by version, followed by all author rebuttals in order by version.

Version 0:

Reviewer comments:

Reviewer #1

(Remarks to the Author)
NATs are a large family of enzymes that catalyze the N-terminal acetylation. In eukaryotes, five different ribosome-associated NATs (NatA, NatB, NatC, NatD, and NatE) have been described that cotranslationally acetylate the N termini of proteins. These NATs differ in the composition of their subunits and display different substrate specificities. In the manuscript, Alves, Knejski and colleagues revealed structural basis of cotranslational N-acetylation by NatB. This work follows on from the authors' series of studies analyzing factors involved in cotranslational processes, including NAC. This is an excellent piece of research, providing a detailed analysis of how NatB binds to the ribosome and, consequently, carries out N-acetylation, including its relationship with NAC. To be honest, I was not particularly surprised by the findings and insights presented in this manuscript, but there is no doubt that it is well worth publishing.

Minor comment:
The competition with other NATs that the authors have already investigated (Fig.4) is an interesting point. If proteomics data on cellular abundance is available, mentioning it in the introduction and/or discussion section would likely satisfy the readers.

Reviewer #2

(Remarks to the Author)
Alves et al. performed a cryo-EM analysis of the Xbp1u stalled 80S ribosome bound with the NatB and NAC complexes and discussed the mechanism by which NatB acetylates the N-terminus of nascent polypeptides. While usual Nat complexes like NatA acetylate the N-terminus of the nascent chain after removal of the fist Met by MetAP1, NatB acetylates the N-terminal Met of the nascent chain. NatB binds near the ribosomal exit tunnel together with NAC. Interactions with ribosome are not only by the auxiliary subunit Naa25 but also by the catalytic subunit Naa20, which differs from the NatA, where the ribosome interactions are only made by its auxiliary subunit. The interactions identified in the cryo-EM structure were thoroughly validated through in vitro and cellular mutational experiments. Using AlphaFold, the authors predicted the interaction between Naa25 of NatB and the UBA domain of NAC, which was subsequently confirmed by mutational experiments.
This study is the first to report a complex that performs N-terminal acetylation without removal of the initiator methionine, making it highly significant for the field. Extensive validation experiments using mutants were conducted, highlighting the overall excellence of the study.

I have only minor comments.
1. In the cryo-EM sample, the RNC complex has the substrate nascent peptide subject to acetylation. Is the N-terminus of this peptide visible in the active site of Naa20? If it is observed, the authors should describe the interactions. If not, they should provide a rationale for why it was not visible. In either case, the mechanism for the peptide sequence specificity achieved by NatB should be discussed.

2. I think it is useful to compare with the reported NatB structures without ribosome to see if there are any conformational changes.

3. The colors of Naa20 and Naa25 in Fig. 1 are too similar to distinguish and should be improved, particularly in Fig. 1b.

4. Is it possible for NatB to coexist with MetAP1 on the ribosome with NAC? If so, this point should be discussed.

5. The image resolution of Supplementary Figure 3 is poor, making the alignment difficult to see. It should be improved.

6. Regarding the Methods section, the preparation of cryo-EM samples, particularly the assembly of the RNC complex, is complicated. It would be helpful to describe the overview of the cryo-EM sample preparation in the main text.

7. In addition, the rationale for using GADD34 Δ240 should be described in the Methods or the main text.

8. The used mRNA has EMCV IRES, eGFP, SUMO, and Xbp1u. Presenting the mRNA architecture in a schematic figure would improve clarity.

9. Lines 406 and 421 in Methods. "$MgCl_2$" appears to be garbled and should be corrected.

10. Supplementary Table 1. Superscription should be used for Å2, Mg2+, and Zn2+.

Reviewer #3

(Remarks to the Author)
General overall assessment:
This work is interesting with novel with important findings to understand the co-translational mechanisms of N-terminal acetylation. Although perhaps somewhat limited in size/scope. Regarding data validity and significance, some parts lack a description of how the data shown was backed by independent replica experiments. This should be included, and replica experiments should be shown in supplemental or source data file.

Specific comments (major: 4-7, minor: 1-3 and 8-10):

1. Please consider aligning nomenclature of key terms with the referenced recent review literature.

2. Abstract: Perhaps consider slightly rewriting the sentence containing 2x "site".

3. Figure legends are somewhat scarce on information, some more methodological/design information could be useful for preciseness and improved readability.

4. Several figure data do not have any information on number of replicate experiments and statistics. In general, experiments should be repeated 3 times, and all data (independent setups) should be included in supplemental and information on reproducibility included in figure legend. At least valid for Fig. 1a, b, d, e and Fig. 3c, d, f and Figure 4b, c, d, e, f.

5. It would be good to see full size WBs overlayed with marker in source data. For Naa25 for example, it is a bit challenging to assess the band size and distinguish from the unspecific(+) band, and it seems like there might be a slight variation across figure (or alternatively slight inaccuracy of positioning the 115 kDa marker label).

6. In Fig. 3d and f: Please assess endogenous Naa25 and Naa20 levels, respectively, to enable analysis of contributions from exogenous versus endogenous NatB subunits.

7. In Fig. 4b: Please include NACalpha detection to verify equal levels of NACalpha WT vs mutants.

8. In the Discussion, please briefly discuss the potential role of NAC in directing also NatC-mediated N-terminal acetylation, and thereby ALL co-translational acetylation in human cells (for example based on NAA35 features).

9. The authors write: "A key distinction of NatB is the additional ribosome contact contributed by its catalytic subunit Naa20 near the tunnel exit (Fig. 2c, 3e and 5d). By contrast, the catalytic subunits of NatA/E (Naa10 and Naa50) are positioned further from the exit, consistent with the requirement for MetAP1 and NMTs to access the nascent N terminus before NatA/E15." Is there perhaps anything in their data along these lines which could potentially provide an explanation to the fact that NatB is the NAT with the highest NtAc degree of its substrates (100% or close to 100%)? (to potentially be descussed in Discussion?).

10. References: Could consider including more literature.

Open Access This Peer Review File is licensed under a Creative Commons Attribution 4.0 International License, which permits use, sharing, adaptation, distribution and reproduction in any medium or format, as long as you give appropriate credit to the original author(s) and the source, provide a link to the Creative Commons license, and indicate if changes were made.
In cases where reviewers are anonymous, credit should be given to 'Anonymous Referee' and the source.
The images or other third party material in this Peer Review File are included in the article's Creative Commons license, unless indicated otherwise in a credit line to the material. If material is not included in the article's Creative Commons license and your intended use is not permitted by statutory regulation or exceeds the permitted use, you will need to obtain permission directly from the copyright holder.
To view a copy of this license, visit https://creativecommons.org/licenses/by/4.0/

We thank all reviewers for their constructive comments according to which we revised and improved the manuscript. The list of specific revisions is provided in point-by-point answers below.

Reviewer #1 (Remarks to the Author):

NATs are a large family of enzymes that catalyze the N-terminal acetylation. In eukaryotes, five different ribosome-associated NATs (NatA, NatB, NatC, NatD, and NatE) have been described that cotranslationally acetylate the N termini of proteins. These NATs differ in the composition of their subunits and display different substrate specificities. In the manuscript, Alves, Knejski and colleagues revealed structural basis of cotranslational N-acetylation by NatB. This work follows on from the authors' series of studies analyzing factors involved in cotranslational processes, including NAC. This is an excellent piece of research, providing a detailed analysis of how NatB binds to the ribosome and, consequently, carries out N-acetylation, including its relationship with NAC. To be honest, I was not particularly surprised by the findings and insights presented in this manuscript, but there is no doubt that it is well worth publishing.

Minor comment:
The competition with other NATs that the authors have already investigated (Fig.4) is an interesting point. If proteomics data on cellular abundance is available, mentioning it in the introduction and/or discussion section would likely satisfy the readers.

As suggested, we now mention the cellular concentrations of the different NATs in the Discussion section and added the corresponding reference (Kulak et al., 2014).

Reviewer #2 (Remarks to the Author):

Alves et al. performed a cryo-EM analysis of the Xbp1u stalled 80S ribosome bound with the NatB and NAC complexes and discussed the mechanism by which NatB acetylates the N-terminus of nascent polypeptides. While usual Nat complexes like NatA acetylate the N-terminus of the nascent chain after removal of the fist Met by MetAP1, NatB acetylates the N-terminal Met of the nascent chain. NatB binds near the ribosomal exit tunnel together with NAC. Interactions with ribosome are not only by the auxiliary subunit Naa25 but also by the catalytic subunit Naa20, which differs from the NatA, where the ribosome interactions are only made by its auxiliary subunit. The interactions identified in the cryo-EM structure were thoroughly validated through in vitro and cellular mutational experiments. Using AlphaFold, the authors predicted the interaction between Naa25 of NatB and the UBA domain of NAC, which was subsequently confirmed by mutational experiments.
This study is the first to report a complex that performs N-terminal acetylation without

removal of the initiator methionine, making it highly significant for the field. Extensive validation experiments using mutants were conducted, highlighting the overall excellence of the study.

I have only minor comments.
1. In the cryo-EM sample, the RNC complex has the substrate nascent peptide subject to acetylation. Is the N-terminus of this peptide visible in the active site of Naa20? If it is observed, the authors should describe the interactions. If not, they should provide a rationale for why it was not visible. In either case, the mechanism for the peptide sequence specificity achieved by NatB should be discussed.

The N-terminus of the nascent chain was not resolved within the substrate-binding pocket, likely due to its flexibility. We now explicitly note this in the Results section. As suggested, we also clarify the basis of NatB peptide sequence specificity in the Introduction of the revised manuscript.

2. I think it is useful to compare with the reported NatB structures without ribosome to see if there are any conformational changes.

We thank the reviewer and appreciate the suggestion. There is no major conformational change between ribosome-bound and ribosome-free NatB. This point is now described in the Discussion.

3. The colors of Naa20 and Naa25 in Fig. 1 are too similar to distinguish and should be improved, particularly in Fig. 1b.

We agree with this comment and, as suggested, have revised the color scheme by strengthening the color of NAA20 and using a lighter shade for NAA25 to further improve contrast. These changes have been implemented in all figures.

4. Is it possible for NatB to coexist with MetAP1 on the ribosome with NAC? If so, this point should be discussed.

The MetAP1 docking site appears to remain accessible on ribosomes bound by NAC and NatB. We discuss this point in the Discussion section of the revised manuscript.

5. The image resolution of Supplementary Figure 3 is poor, making the alignment difficult to see. It should be improved.

As suggested, we improved Supplementary Figure 3.

6. Regarding the Methods section, the preparation of cryo-EM samples, particularly the assembly of the RNC complex, is complicated. It would be helpful to describe the overview of the cryo-EM sample preparation in the main text.

As suggested, we provide details of how the RNC complex was reconstituted in the main text of the revised manuscript.

7. In addition, the rationale for using GADD34 Δ240 should be described in the Methods or the main text.

As suggested, we have clarified the rationale for using GADD34 Δ240 in the *in vitro* translation reaction and included this explanation in the methods section.

8. The used mRNA has EMCV IRES, eGFP, SUMO, and Xbp1u. Presenting the mRNA architecture in a schematic figure would improve clarity.

As suggested, a schematic figure of mRNA architecture is now shown in the Supplementary Fig. 6.

9. Lines 406 and 421 in Methods. “MgCl□” appears to be garbled and should be corrected.

This was likely a PDF conversion problem. The text file appears correct.

10. Supplementary Table 1. Superscription should be used for Å2, Mg2+, and Zn2+.

As suggested, we applied superscription and updated the table.

Reviewer #3 (Remarks to the Author):

General overall assessment:
This work is interesting with novel with important findings to understand the co-translational mechanisms of N-terminal acetylation. Although perhaps somewhat limited in size/scope. Regarding data validity and significance, some parts lack a description of how the data shown was backed by independent replica experiments. This should be included, and replica experiments should be shown in supplemental or source data file.

Specific comments (major: 4-7, minor: 1-3 and 8-10):

1. Please consider aligning nomenclature of key terms with the referenced recent review literature.

As suggested, we changed the nomenclature of some key terms. Throughout text and figures, we changed ‘N-acetylation’ into ‘Nt-acetylation’, and ‘Naa20, Naa25 etc’ into ‘NAA20, NAA25 etc’.

2. Abstract: Perhaps consider slightly rewriting the sentence containing 2x “site”.

As suggested, we corrected this sentence in the abstract.

3. Figure legends are somewhat scarce on information, some more methodological/design information could be useful for preciseness and improved readability.

As suggested, we now provide more technical information of the experiments in the figure legends.

4. Several figure data do not have any information on number of replicate experiments and statistics. In general, experiments should be repeated 3 times, and all data (independent setups) should be included in supplemental and information on reproducibility included in figure legend. At least valid for Fig. 1a, b, d, e and Fig. 3c, d, f and Figure 4b, c, d, e, f.

Information on the number of replicates of each experiment is provided in the figure legends, the methods, and the Nature Portfolio Reporting Summary linked to this article. Raw data of experiments are provided in the attached Source data file (according to editorial guidelines).

5. It would be good to see full size WBs overlayed with marker in source data. For Naa25 for example, it is a bit challenging to assess the band size and distinguish from the unspecific(+) band, and it seems like there might be a slight variation across figure (or alternatively slight inaccuracy of positioning the 115 kDa marker label).

Uncropped gels and blots are provided in the attached Source data file.

6. In Fig. 3d and f: Please assess endogenous Naa25 and Naa20 levels, respectively, to enable analysis of contributions from exogenous versus endogenous NatB subunits.

As suggested, we added the control data without ectopic expression in Fig. 3d and f.

7. In Fig. 4b: Please include NACalpha detection to verify equal levels of NACalpha WT vs mutants.

NACα levels of purified WT and mutant NAC variants used in Fig. 4 are shown in Supplementary Fig. 5b.

8. In the Discussion, please briefly discuss the potential role of NAC in directing also NatC-mediated N-terminal acetylation, and thereby ALL co-translational acetylation in human cells (for example based on NAA35 features).

The NatB residues that mediate interaction with the NACα UBA domain (NAA25 L176, R179, M180) are not conserved in NAA35, suggesting that NatC may not engage NAC in the same manner. However, we cannot exclude that NAC interacts

with the trimeric NatC complex through a distinct interface. We now note in the Discussion that the ribosome-binding mechanism of NatC, and any potential role of NAC in this context, remain unknown.

9. The authors write: “A key distinction of NatB is the additional ribosome contact contributed by its catalytic subunit Naa20 near the tunnel exit (Fig. 2c, 3e and 5d). By contrast, the catalytic subunits of NatA/E (Naa10 and Naa50) are positioned further from the exit, consistent with the requirement for MetAP1 and NMTs to access the nascent N terminus before NatA/E15.” Is there perhaps anything in their data along these lines which could potentially provide an explanation to the fact that NatB is the NAT with the highest NtAc degree of its substrates (100% or close to 100%)? (to potentially be discussed in Discussion?).

We thank the reviewer for this thoughtful suggestion. The high degree of N-terminal acetylation observed for NatB substrates is indeed an interesting feature. However, our current data do not directly address the mechanistic basis for this observation, and we therefore cannot provide a definitive explanation. While the proximity of the NAA20 active site to the tunnel exit could in principle contribute to efficient substrate capture, this remains speculative, given that also NatD’s active site is located close to the tunnel exit. To avoid overinterpretation, we have chosen not to elaborate further on this point in the Discussion.

10. References: Could consider including more literature.

We agree that additional references will improve the manuscript and incorporated further relevant literature in the revised version.
